# Mol­ecular and crystal structures of six poly(arylsulfin­yl)- and poly(aryl­sulfan­yl)fer­ro­cenes

**DOI:** 10.1107/S2053229624009318

**Published:** 2024-10-04

**Authors:** Tobias Blockhaus, Karlheinz Sünkel

**Affiliations:** a Chemistry, Ludwig-Maximilians-University Munich, Butenandtstrasse 5-13, Munich, D-81377, Germany; University of Melbourne, Australia

**Keywords:** crystal structure, sul­fan­ylfer­ro­cene, sulfinyl­fer­ro­cene, Hirshfeld analysis, iron, inter­action energy

## Abstract

Starting from (*p*-tolyl­sulfin­yl)fer­ro­cene (**1**), a mixture of the complete series [CpFe{C_5_H_5*–n*_(SOTol-*p*)_*n*_}] (*n* = 2–4) (**2**–**4**) in all regioisomers was obtained. After chromatographic separation, crystals of 1,2-bis­[(4-methyl­benzene)­sulfin­yl]fer­ro­cene, **2a**, and 1,3-bis­[(4-methyl­benzene)­sulfin­yl]fer­ro­cene, **2b**, both [Fe(C_5_H_5_)(C_19_H_17_O_2_S_2_)], as well as of 1,2,3-tris­[(4-methyl­benzene)­sulfin­yl]fer­ro­cene, [Fe(C_5_H_5_)(C_26_H_23_O_3_S_3_)], **3a**, and 1,2,3,4-tetra­kis­[(4-methyl­benzene)­sul­fin­yl]fer­ro­cene ethyl acetate 0.75-solvate, [Fe(C_5_H_5_)(C_33_H_29_O_4_S_4_)]·0.75C_4_H_8_O_2_, **4**, could be isolated. Their mol­ecular and crystal structures are compared with each other and also with the so far un­reported structures of related 1,2-bis­(phenyl­sulfan­yl)fer­ro­cene, [Fe(C_5_H_5_)(C_17_H_13_S_2_)], **5**, and 1,2,3,4-tetra­kis­(phenyl­sulfan­yl)fer­ro­cene, [Fe(C_5_H_5_)(C_29_H_21_S_4_)], **6**. In all the sulfinyl structures, the O atoms of the S=O groups are in equatorial positions, except for that in tetrasubstituted **4**. All the arene rings of these com­pounds (except for one ring in **4**) are in axial positions directed away from the Fe atom, mostly in a near perpendicular orientation with respect to the plane of the cyclo­penta­di­en­yl ring. The main inter­molecular inter­actions in the crystals are C—H⋯H—C, C—H⋯π and C—H⋯O, while C—H⋯S inter­actions are much less important, except for tetra­sul­fan­yl com­pound **6**. π–π inter­actions (intra­molecular) are only important in com­pound **3a**. Hirshfeld analysis shows that dispersion terms are dominant for the inter­action energies of all six com­pounds. In general, the calculated total inter­action energies increase with increasing number of substituents and are higher for the sulfinyl than for the sul­fan­yl groups.

## Abstract 2

The structures of four polysulfinyl- and two polysul­fan­ylfer­ro­cenes were examined. Nonclassical C—H⋯*X* (*X* = O, S and C) hydrogen bonds have a large influence on most of the individual crystal structures, with different importance for the sulfinyl and sul­fan­yl com­pounds. Chalcogen O⋯O, S⋯S or S⋯O bonding is not significant, except for one com­pound, and π–ring inter­actions are important for only one com­pound.

## Introduction

(*R*_S_)-(*p*-Tolyl­sulfinyl)­fer­ro­cene [CpFe(C_5_H_4_SOTol-*p*)] (**1**) was first reported by Rebiere *et al.* (1990 ▸). It was obtained by treatment of li­thio­fer­ro­cene with the Andersen reagent, *i.e.* (+)-(*R*_S_,1*S*)-menthyl *p*-toluenesulfinate (Andersen, 1964 ▸). An alternative approach, also introduced by the group of Kagan, used the enanti­oselective oxidation of the corresponding ferrocenyl sulfide (Diter *et al.*, 1994 ▸). Treating this com­pound with lithium diiso­propyl­amide (LDA) led to diastereoselective *ortho*-li­thia­tion (Rebière *et al.*, 1993 ▸) and, after quenching with appropriate electrophiles, a selective synthesis of planar–chiral fer­ro­cenes was possible (Ferber & Kagan, 2007 ▸; Schaarschmidt & Lang, 2013 ▸). While numerous unsymmetrically disubstituted and, therefore, planar–chiral fer­ro­cenes have been reported, there are very few reports on disubsti­tu­ted fer­ro­cenes, [CpFe(C_5_H_3_*R*_2_)], with two identical substituents carrying the same chirality on the α-atom of the sub­stituent: *R* = CHMe(OH) (Moïse & Mugnier, 1972 ▸), CHPh(OAc), CHPh(N_3_) and CHPh(NH_2_) (Fukuzawa & Suzuki, 2006 ▸), CHMe(PPh_2_BH_3_) (Fukuzawa *et al.*, 2000 ▸), and, more recently, *R* = SO(*t*-Bu) or SO(Tol-*p*) (Wen *et al.*, 2022 ▸). The latter article described the synthesis of (*S*_S_,*S*_S′_)-[CpFe{C_5_H_3_(SOTol-*p*)}_2_-1,2)] (**2a**). Apparently, no metallocenes with more than two aryl­sulfinyl substituents have been reported so far. A search in the Cambridge Structural Database (CSD, accessed on March 10, 2024; Groom *et al.*, 2016 ▸) shows 35 entries for the search mask ‘[CpFe{C_5_(SOPh)}]’, including three sulfone com­pounds. Nine of the 35 contained a {C_5_H_4_SOPh} ring, while 26 were 1,2-disubstituted. None con­tains more than one sulfinyl substituent and no 1,3-disubstituted structure was reported.

Structurally related to aryl­sulfinyl groups are aryl­sul­fan­yl groups, which create inter­esting electrical properties on the mol­ecules to which they are bound. It was found that ‘phenyl­thiol substituents attached to aromatic cores result in a reduction of the HOMO–LUMO gap’ (HOMO is the highest occupied mol­ecular orbital and LUMO is the lowest unoccupied mol­ecular orbital) (Gingras *et al.*, 2006 ▸; Deng *et al.*, 2017 ▸). Previously, we described the synthesis of the aryl­sul­fan­ylfer­ro­cenes [CpFe{C_5_H_5*–n*_(SPh)_*n*_}] (*n* = 1–5) (Blockhaus *et al.*, 2019 ▸) and reported the crystal structure of the penta­substituted com­pound. We found it worthwhile to study the synthesis of poly(aryl­sulfin­yl)fer­ro­cenes [CpFe{C_5_H_5*-n*_(SOAr)_*n*_}] with *n* ≥ 2 and compare their crystal and mol­ecular structures with the corresponding poly(aryl­sulfan­yl)fer­ro­cenes. Scheme 1 shows the com­pounds discussed in the present study. To the best of our knowledge, there is only one systematic study comparing organic sulfides and sulfoxides with respect to the inter­molecular inter­actions in the crystal (Zhou *et al.*, 2021 ▸).
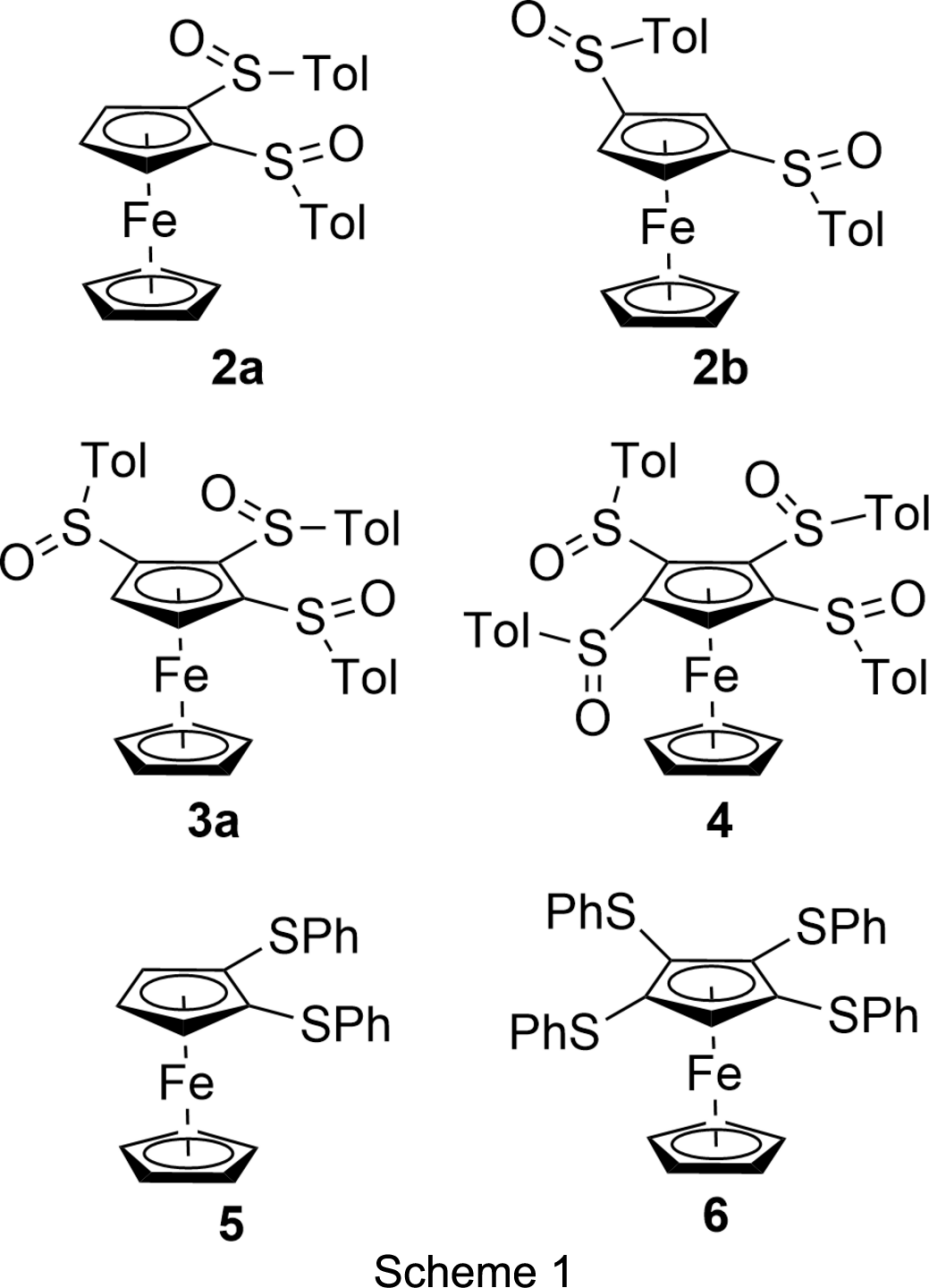


## Experimental

### Synthesis and crystallization

Reactions were carried out under an argon atmosphere using standard Schlenk techniques. The anhydrous solvents and LDA (1.0 *M* in THF/hexane, Sigma–Aldrich) were used as provided. Andersen’s reagent was prepared according to the literature (Andersen, 1964 ▸)

Column chromatography was performed on silica gel (Acros Organics) using petroleum ether (PE), diethyl ether (Et_2_O), di­chloro­methane (CH_2_Cl_2_) or ethyl acetate (EA), or mixtures thereof, as eluents.

#### Synthesis of (*S*_S_)-(*p*-tolyl­sulfin­yl)fer­ro­cene, 1

A solution of fer­ro­cene (6.52 g, 35.1 mmol) and KO*t*-Bu (0.47 g, 4.21 mmol) in THF (100 ml) was treated at −78 °C with *tert*-butyl­lithium solution (22 ml), with stirring for 30 min at −78 °C and 30 min at room tem­per­a­ture. Then, at −78 °C, Andersen’s reagent (10.32 g, 35.05 mmol) was added and stirring continued for 18 h. After evaporation of the solvents *in vacuo*, the residue was placed on top of a silica-gel column and extracted with di­chloro­methane (5 × 100 ml). After re­moval of the solvents *in vacuo*, the desired product was obtained after chromatography on silica gel, using a 1:1 (*v*/*v*) PE/Et_2_O mixture as eluent (yield: 6.56 g, 20.3 mmol, 58%). For the ^1^H NMR spectrum, see Fig. S17 of the supporting information.

#### Reaction of 1 with LDA and Andersen’s reagent

A solution of **1** (2.400 g, 7.41 mmol) in THF (75 ml) was treated at −78°C with 1.0 *M* LDA solution (8.90 ml, 8.90 mmol) with stirring for 45 min. Then, after addition of solid Andersen reagent (2.620 g, 8.90 mmol) with continuous stirring, the reaction mixture was warmed gradually to room tem­per­a­ture (20 °C) within 16 h. After evaporation of the obtained suspension, the residue was taken up in the minimum amount of ethyl acetate and placed on top of a silica-gel column. Repeated chromatography was necessary to afford separations of the products.

**(1*S*_S_,2*S*_S_)-1,2-Bis[(4-methyl­benzene)­sulfin­yl]fer­ro­cene (**2a**).**^1^H NMR (400 MHz, CDCl_3_, Fig. S8): δ 7.46 (*m*, 4H), 7.22 (*m*, 2H), 7.10 (*m*, 2H), 4.91 (*m*, 1H), 4.55 (*s*, 5H), 4.48 (*m*, 1H), 4.23 (*m*, 1H), 2.40 (*s*, 3H), 2.32 (*s*, 3H); ^13^C{^1^H} NMR (101 MHz, CDCl_3_, Fig. S9): δ 141.8, 141.1, 129.7, 125.3, 125.2, (87.8, assignment dubious), 72.2, 70.8, 67.2, 21.6, 21.5; HRMS (+p ESI): *m*/*z* 463.04871 (*M* + H^+^; calculated for C_24_H_23_O_2_FeS_2_: 463.04891); IR (ATR, cm^−1^): ν(SO) 1733, 1714.

**(1*S*_S_,3*S*_S_)-1,3-Bis[(4-methyl­benzene)­sulfin­yl]fer­ro­cene (**2b**).**^1^H NMR (270 MHz, CDCl_3_, Fig. S10): δ 7.50 (*m*, 2H), 7.47 (*m*, 2H), 7.28 (*m*, 2H), 7.26 (*m*, overlapped with solvent), 4.80 (*m*, 1H), 4.78 (*m*, 1H), 4.54 (*s*, 5H), 4.49 (*m*, 1H), 2.39 (*s*, 6H); ^13^C{^1^H} NMR (101 MHz, CDCl_3_, Fig. S11): δ 142.0, 141.9, 141.8, 130.03, 129.99, 124.63, 124.55, 86.4, 96.3, 72.0, 69.3, 67.5, 66.2, 21.6; HRMS (+p ESI): *m*/*z* 501.00495 (*M* + K^+^, calculated for C_24_H_22_KO_2_FeS_2_: 501.00481); IR (ATR, cm^−1^): ν(SO) 1736, 1718.

**(1*S*_S_,2*S*_S_,3*S*_S_)-1,2,3-Tris[(4-methyl­benzene)­sulfin­yl]fer­ro­cene (**3a**).**^1^H NMR (270 MHz, CDCl_3_ Fig. S12): δ 7.48 (*m*, 2H), 7.25 (*m*, overlapped with solvent), 7.13 (*m*, 2H), 6.96–6.86 (*m*, 6H), 5.13 (*m*, 1H), 4.79 (*s*, 5H), 4.36 (*m*, 1H), 2.40 (*s*, 3H), 2.27 (*s*, 3H), 2.25 (*s*, 3H); ^13^C{^1^H} NMR (101 MHz, CD_2_Cl_2_, Fig. S13): δ 143.7, 142.7, 142.5, 141.6, 141.3, 139.9, 130.1, 129.70, 129.66, 125.8, 125.5, 125.4, 98.6, 97.1, 95.4, 74.5, 70.8, 68.0, 21.6, 21.41, 21.39; HRMS (+p ESI): *m*/*z* 601.06268 (*M* + H^+^, calculated for C_31_H_29_O_3_FeS_3_: 601.06285); IR (ATR, cm^−1^): ν(SO) 1736.

**(1*S*_S_,2*S*_S_,3*S*_S_,4*S*_S_)-1,2,3,4-Tetra­kis[(4-methyl­benzene)­sulfin­yl]fer­ro­cene (**4**).**^1^H NMR (400 MHz, CDCl_3_, Fig. S14): δ 7.75 (*m*, 2H), 7.41 (*m*, 2H), 7.21 (*m*, 2H), 7.17 (*m*, 2H), 6.90 (*m*, 2H), 6.80 (*m*, 2H), 6.51 (*m*, 2H), 6.21 (*m*, 2H), 5.16 (*s*, 1H), 4.96 (*s*, 5H), 2.50 (*s*, 3H), 2.26 (*s*, 3H),2.19 (*s*, 3H), 2.16 (*s*, 3H); ^13^C{^1^H} NMR (101 MHz, CDCl_3_, Fig. S15): δ 143.5, 142.8, 141.8, 141.7, 141.6, 141.3, 141.0, 140.8, 140.0, 138.1, 130.4, 129.7, 129.50. 129.48, 129.0, 126.34, 126.25, 125.8, 125.5, 125.0, 98.6, 98.4, 97.3, 94.3, 92.8, 71.9, 21.8, 21.6, 21.5, 21.3; HRMS (+p ESI): *m*/*z* 739.07683 (*M* + H^+^, calculated for C_38_H_35_O_4_FeS_4_: 739.07682).

### Refinement

Some remarks are necessary with regard to the structure of com­pound **4**. The crystals of this com­pound contain in their voids ethyl acetate solvent molecules. One of them is ‘well behaved’, with no sign of disorder, while the other shows a disorder of the kind that the terminal methyl groups are screw-related, *i.e.* the CH_3_CO methyl group coincides with the screw-related (−*x*, *y* + 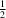, −*z*) OCH_2_CH_3_ methyl group of the next mol­ecule. Since this is chemically impossible, the site-oc­cupancy factor (s.o.f.) was restricted to 0.5. It was also necessary to restrain all bonds within the disordered mol­ecules to be the same as the corresponding bonds of the ordered solvent mol­ecule (five SADI instructions in *SHELXL*). The refinement showed also some problems with the anisotropic displacement parameters of the cyclo­penta­dienyl (Cp) ring of mol­ecule *A* (most likely unresolved disorder, combined with strong librations). To overcome this problem, further restraints were necessary (ISOR and DELU instructions were applied for all five Cp C atoms, *i.e.* the *U^ij^* components were modelled approximately isotropically and rigid-bond restraints were applied). Further crystal data, data collection and structure refinement details are summarized in Table 1 ▸.

## Results and discussion

### Synthesis

We decided to use the original procedure of Kagan, with only slight modifications, for the synthesis of (*S*_S_)-(*p*-tolyl­sulfin­yl)fer­ro­cene [(*S*_S_)-**1**]. We did not check the enanti­omeric purity, as we hoped that necessary purifications would be easier at a later stage. In this context, we also became aware of a report on ‘problems with the accurate determination of the stereochemical outcome’ of such reactions (Han *et al.*, 2018 ▸). We then treated the isolated product with 1.2 equivalents of LDA and 1.2 equivalents of Andersen’s reagent in THF at −78 °C, followed by warming to room tem­per­a­ture (Scheme 2).
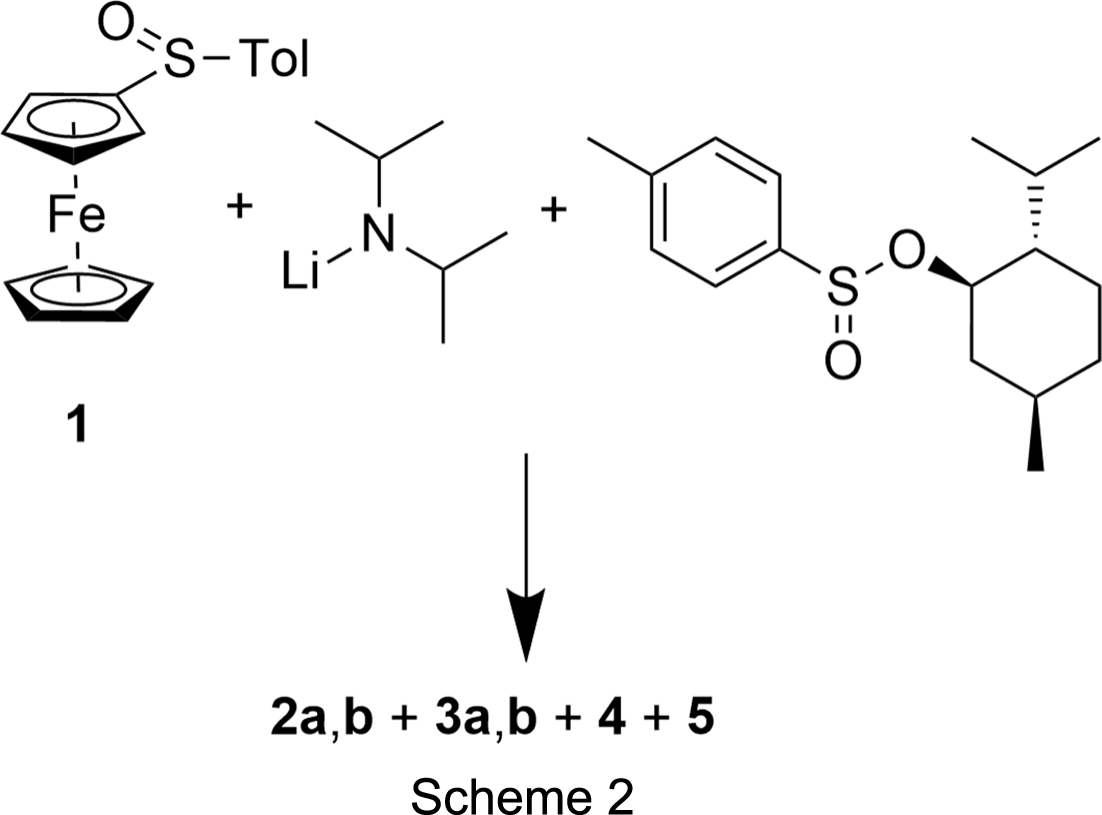


A ^1^H NMR spectrum of the crude product showed a myriad of signals. After several chromatographic separations, regio­isomers **2a** and **2b**, as well as **3a** and **4**, could be isolated in pure forms, albeit in low yields.

The syntheses of the poly(phenyl­sulfan­yl)fer­ro­cenes [CpFe{C_5_H_3_(SPh)_2_-1,2}] (**5**) and [CpFe{C_5_H(SPh)_4_}] (**6**) were re­ported by us previously (Blockhaus *et al.*, 2019 ▸).

### Mol­ecular structures

#### Disubstituted fer­ro­cenes

**Figure 1:**
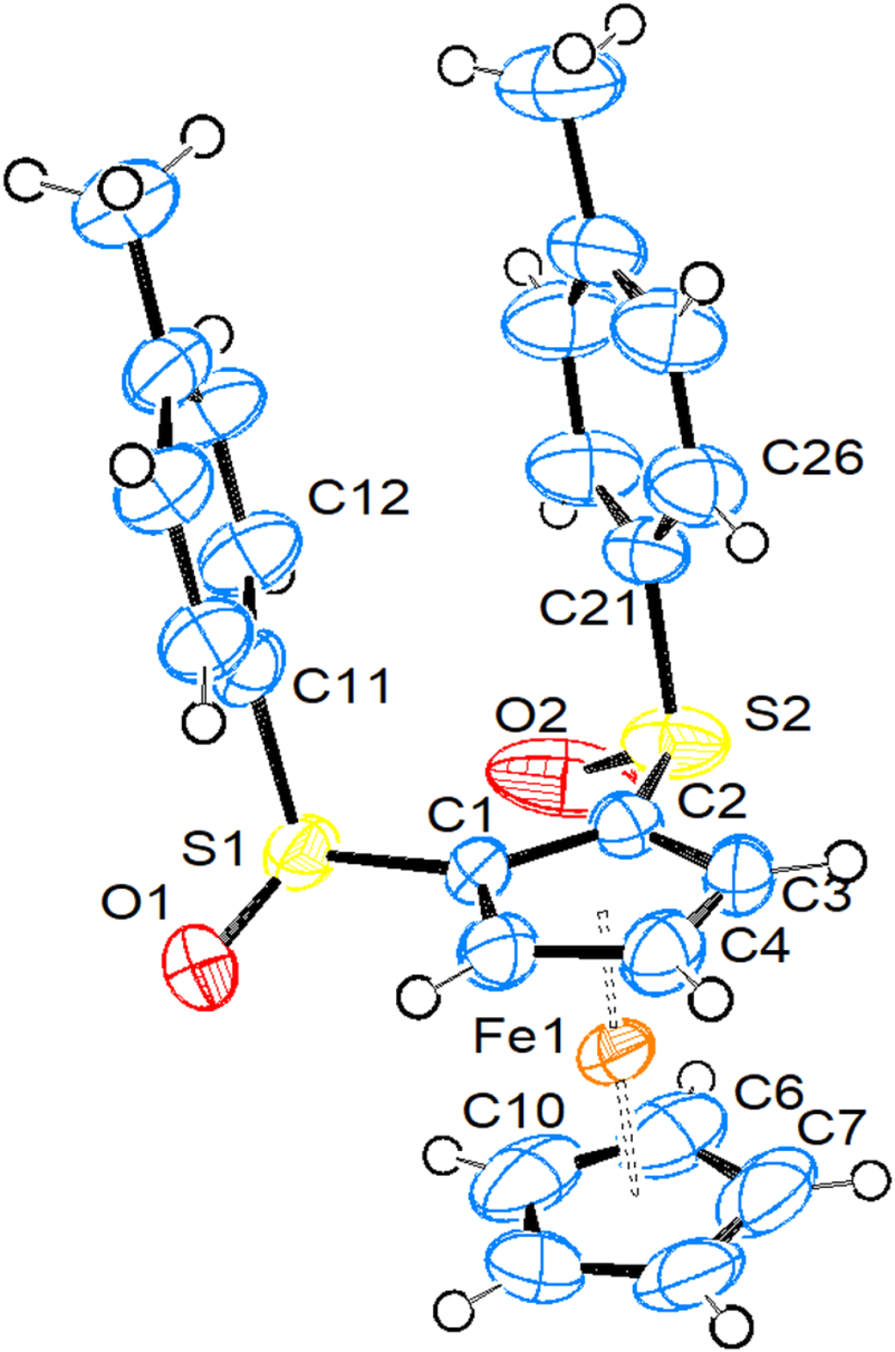
View of the mol­ecular structure of com­pound **2a**. Displacement ellipsoids are drawn at the 50% probability level.

**Figure 2:**
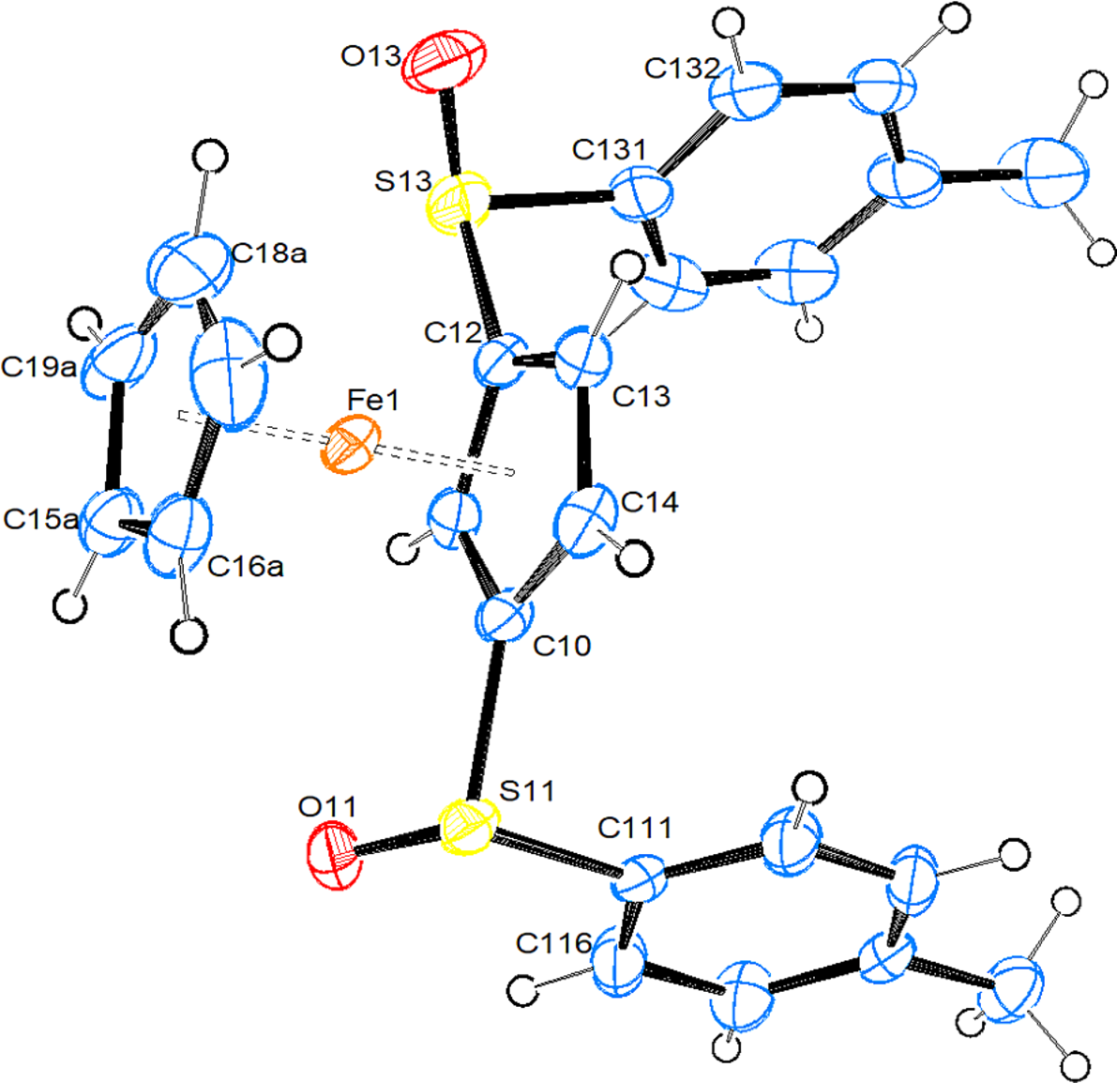
View of the mol­ecular structure of mol­ecule *A* of com­pound **2b**. Displacement ellipsoids are drawn at the 50% probability level.

**(1*S*_S_,2*S*_S_)-1,2-Bis[(4-methyl­benzene)­sulfin­yl]fer­ro­cene (2a) and (1*S*_S_,3*S*_S_)-1,3-bis­[(4-methyl­benzene)­sulfin­yl]fer­ro­cene (2b).**Compound **2a** crystallizes in the monoclinic space group *P*2_1_ with one mol­ecule in the asymmetric unit. Fig. 1 ▸ shows a side view of the mol­ecule (a top view is shown in Fig. S1 of the supporting information). Compound **2b** also crystallizes in the monoclinic space group *P*2_1_, however, with two mol­ecules in the asymmetric unit. Fig. 2 ▸ shows a side view of mol­ecule *A*, while top views of mol­ecules *A* and *B* can be found in the supporting information (Fig. S2).

**Figure 3:**
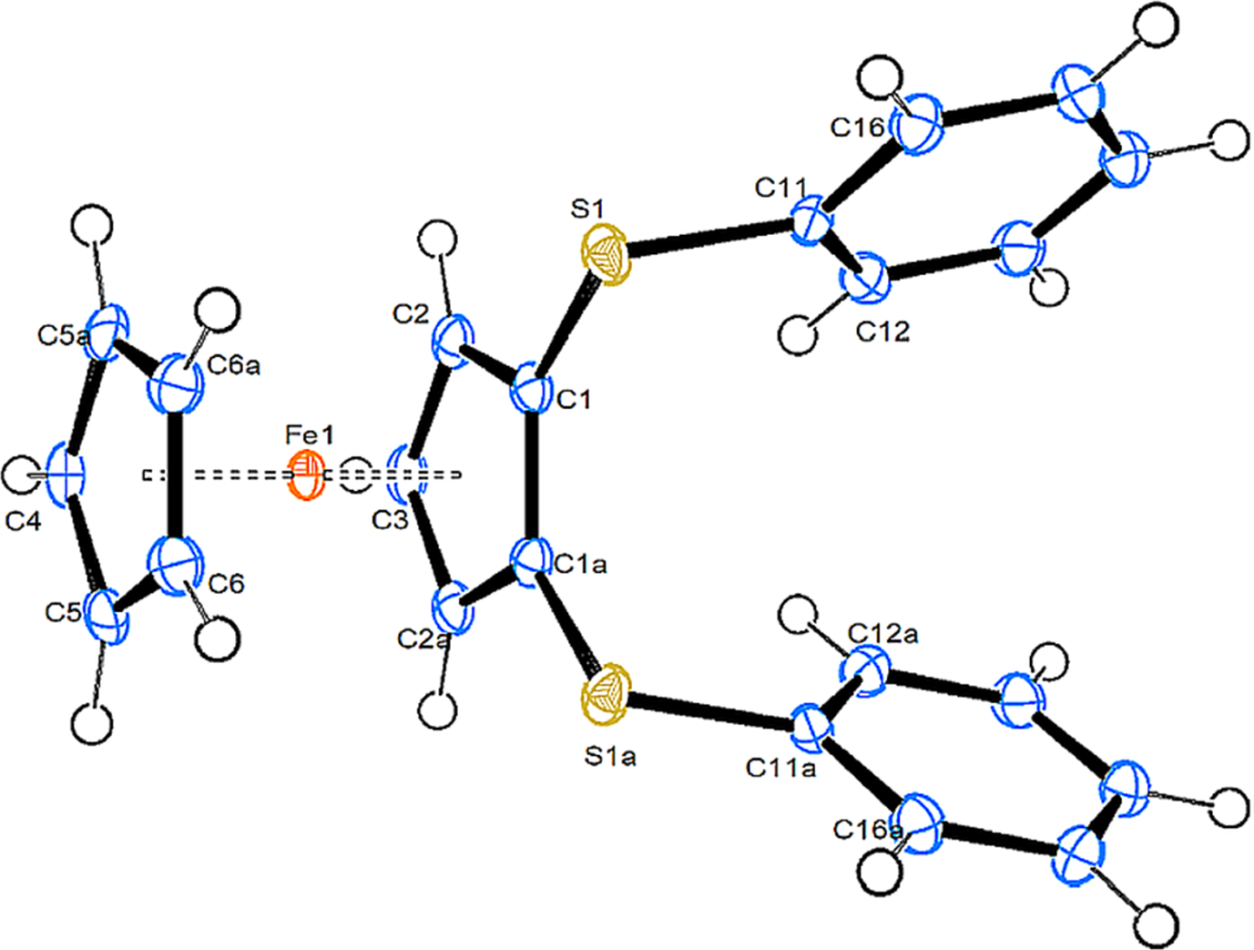
View of the mol­ecular structure of the whole mol­ecule of com­pound **5**. Displacement ellipsoids are drawn at the 50% probability level.

**1,2-Bis(phenyl­sulfan­yl)fer­ro­cene (5).** Compound **5**, [FeCp{C_5_H_3_(SPh)_2_-1,2}], crystallizes in the ortho­rhom­bic space group *Pmn*2_1_, with half a mol­ecule in the asymmetric unit. Fig. 3 ▸ shows a side view of one complete mol­ecule.

The Fe1, C3 and C4 atoms are located on the mirror plane. The Cp rings are exactly eclipsed and all bond parameters occur necessarily pairwise.

Important bond parameters are collected in Table 2 ▸, together with literature data for the mono(tolyl­sulfin­yl)fer­ro­cene (**1**) (CSD refcode VEZPUM) and the 1,2-disubstituted [CpFe{C_5_H_3_(CH_2_NMe_2_)(SOTol-*p*)}] (HEZMIJ) (both in Heinemann *et al.*, 2007 ▸), and [CpFe{C_5_H_3_(CH_2_OH)(SOTol-*p*)}] (TELHOH; Robinson *et al.*, 1996 ▸).

As can be seen from Table 2 ▸, the distances from Fe to the unsubstituted Cp ring are slightly longer than from Fe to the substituted Cp ring. The relative orientation of the Cp rings is close to eclipsed in **1**, **2a** and the CH_2_OH-substituted com­pound TELHOH. The C—S bonds from the Cp rings are slightly shorter in **2a** than in all the other com­pounds, while the S—O bonds in disulfinyl complexes **2a** and **2b** are slightly shorter than in the monosulfinyl com­pounds. The ring C—S bond in **5** is slightly shorter than in the sulfinyl com­pounds, and lies exactly in the arene plane. The O atoms on sulfur are in equatorial positions directed towards the Fe atom in com­pounds **1**, **2a** and **2b** (the Fe⋯O distances are between 3.66 and 3.82 Å, and torsion angles CT—CT—S—O (CT is as defined in Tables 2 ▸ and 3 ▸) are in the range 61–74°), while they are in axial positions directed away from Fe in the remaining two com­pounds (Fe⋯O distance > 4.5 Å and torsion angles CT—CT—S—O in the range 170–176°). The C—S—O angles are all between 103 and 109°, with no recognizable trends. The arene rings on sulfur are always close to being perpendicular with respect to the plane of the Cp ring, spanning a range from *ca* 81° to nearly 90° (in sul­fan­yl com­pound **5**). Whenever the O atom is in an equatorial position, the S—O bond assumes an angle between 13 and 28° with respect to the arene plane, while the S—O bond orients itself nearly perpendicular when the O atom is in an axial position. In all the title com­pounds, the Fe⋯S distance is significantly shorter than the sum of the van der Waals radii (3.80 Å), with the shortest distance being observed for com­pound **5**.

#### Trisubstituted fer­ro­cene

**Figure 4:**
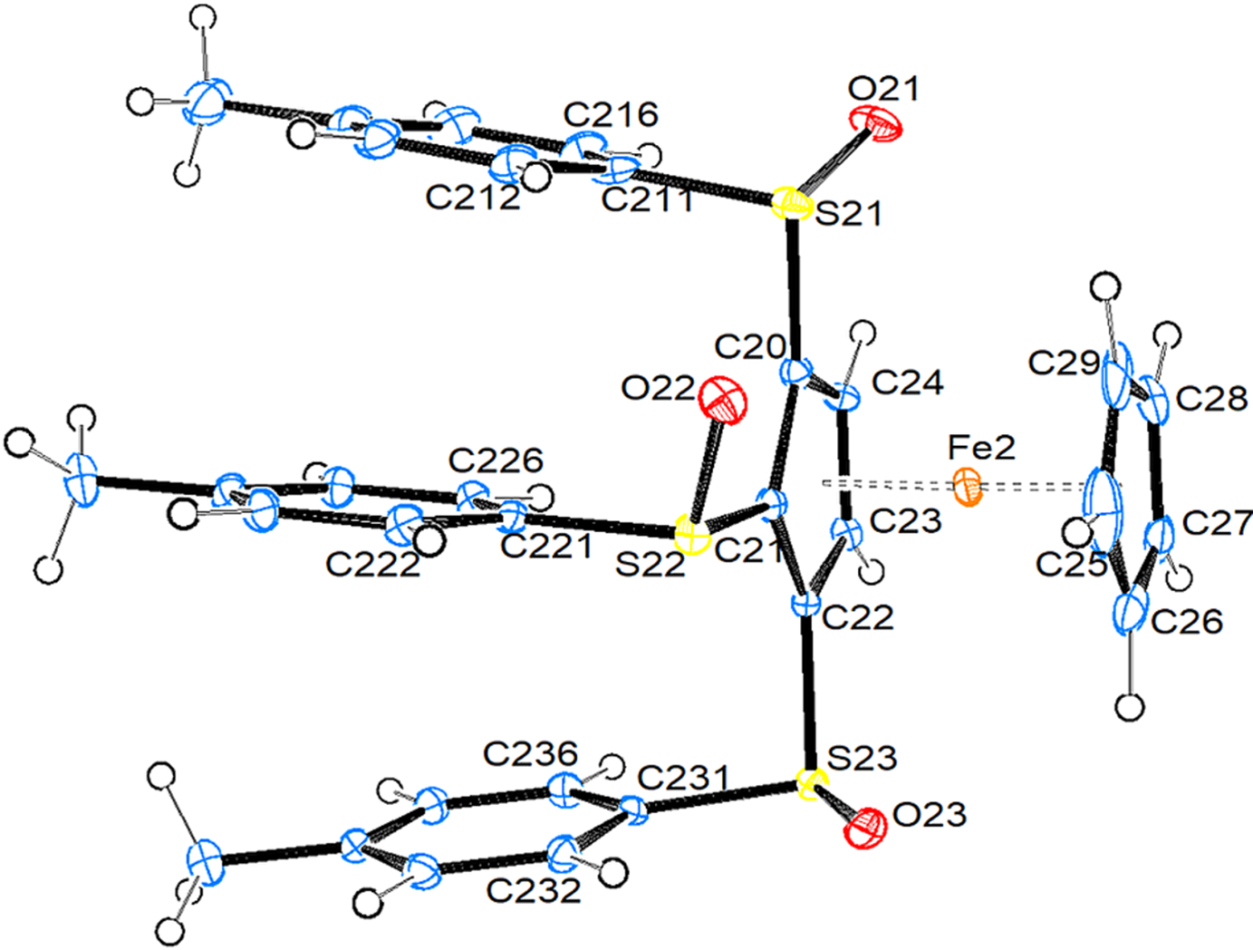
View of the mol­ecular structure of mol­ecule *B* of com­pound **3a**. Displacement ellipsoids are drawn at the 50% probability level.

Compound **3a** crystallizes in the triclinic space group *P*1, with two mol­ecules in the asymmetric unit. Fig. 4 ▸ shows a side view of mol­ecule *B*. Top views of mol­ecules *A* and *B* can be found in the supporting information (Fig. S3). Table 3 ▸ collects important bond parameters for com­pounds **3a**, **4** and **6**.

There is a slight tendency for the distances between Fe and the substituted Cp ring to decrease with increasing degree of substitution, while the distance to the unsubstituted ring remains unchanged. There seems to be no effect of the number of sulfinyl substituents on the relative orientation of the Cp rings. Both mol­ecules of **3a** have nearly perfectly eclipsed Cp rings, as in com­pounds **1** and **2a**. The C—S bonds become gradually longer with increasing degree of substitution, while there is no observable trend in the S—O bond lengths. All O atoms are in equatorial positions (Fe⋯O distances between 3.59 and 3.86 Å and torsion angles CT—CT—S—O in the range 49–84.5°). All arene rings adopt a nearly perpendicular orientation with respect to the plane of the Cp ring, and are close to being parallel to each other. The relative orientation of the S—O bond and the plane of the arene ring spans the whole range from coplanar to nearly perpendicular. Quite inter­estingly, in both mol­ecules, the ring C—S vector of the ‘middle’ bond lies in the plane of the corresponding arene ring, while for the ‘outer’ two C—S bonds, these vectors and the arene planes are at a 70 ± 5° angle. The Fe⋯S distances are well below the sum of the van der Waals radii, spanning, however, a relative large range between 3.319 (1) and 3.441 (1) Å.

#### Tetra­substituted fer­ro­cene

**Figure 5:**
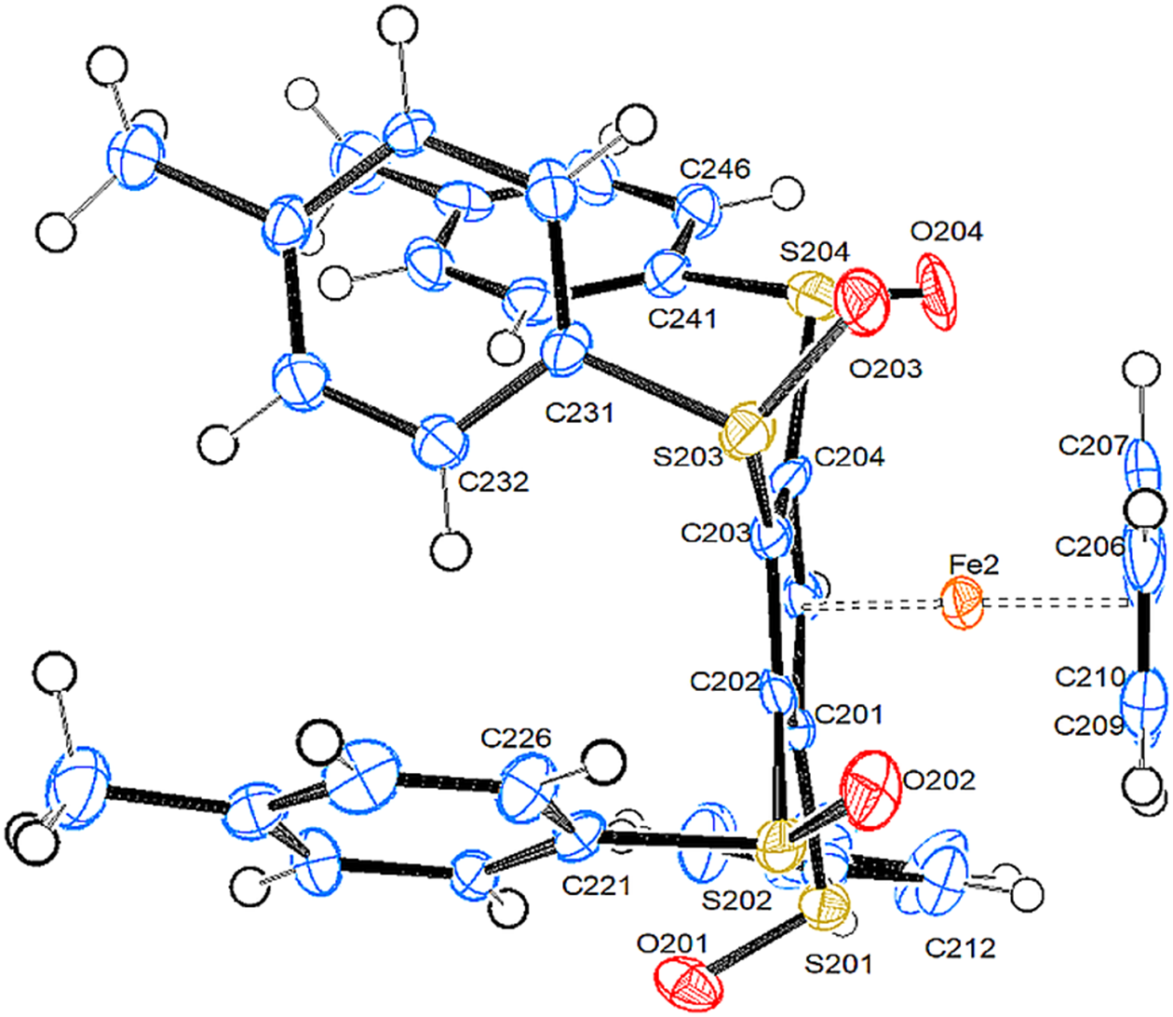
View of the mol­ecular structure of mol­ecule *B* of com­pound **4**. Displacement ellipsoids are drawn at the 50% probability level.

**1,2,3,4-Tetra­kis[(4-methyl­benzene)­sulfin­yl]fer­ro­cene (4)**. Com­pound **4** crystallizes in the monoclinic space group *P*2_1_ with two mol­ecules in the asymmetric unit. In addition, there is one mol­ecule of ethyl acetate, which shows no disorder, and another half mol­ecule of this solvent, which shows disorder. Fig. 5 ▸ shows a side view of mol­ecule *B*. Top views of mol­ecules *A* and *B* can be found in the supporting information (Fig. S4).

The Cp rings are more staggered than in the other com­pounds, and the substituted ring is closer to the Fe atom than in the less substituted complexes. Compound **4** is the only one of the studied com­pounds where one sulfinyl O atom is in an axial position. The other three O atoms are – as usual – in equatorial positions. However, in literature com­pounds HEZMIJ and TELHOH, the O atom is also in an axial position. As these com­pounds are only disubstituted fer­ro­cenes with only one sulfinyl substituent, it becomes clear that shifting the O atom into an axial position is not a consequence of steric congestion in compound **4**. The Fe⋯S distances are – as in all other com­pounds described in this study – well below the sum of the van der Waals radii, spanning a range from 3.302 (2) to 3.407 (2) Å. All arene rings – except for one in mol­ecule *A* – are close to being perpendicular with respect to the plane of the Cp ring. In contrast to com­pound **3a**, there is no tendency of the arene rings to orient themselves parallel to each other.

**Figure 6:**
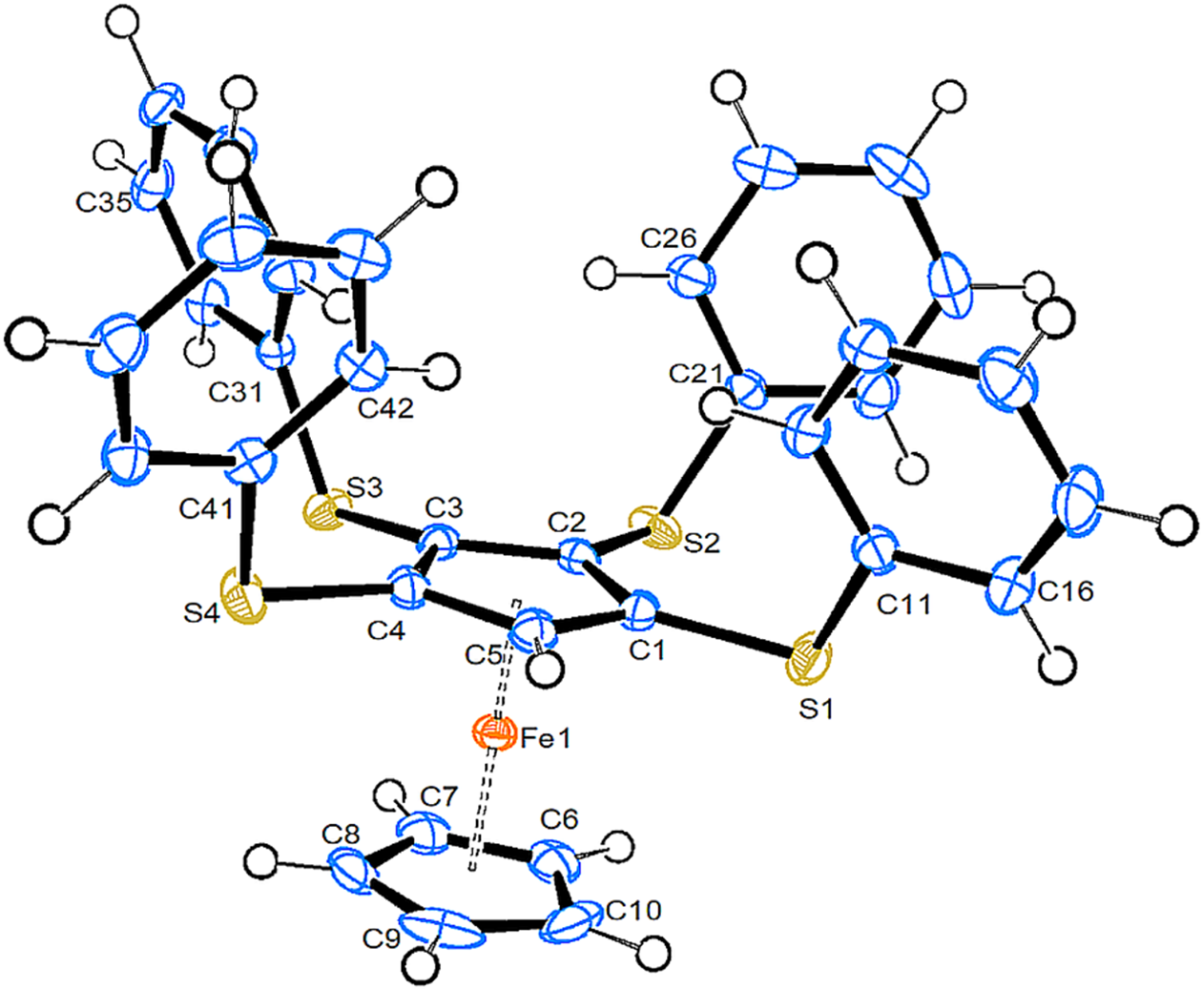
View of the mol­ecular structure of com­pound **6**. Displacement ellipsoids are drawn at the 50% probability level.

**1,2,3,4-Tetra­kis(phenyl­sulfan­yl)fer­ro­cene (6)**. Compound **6** crystallizes in the triclinic space group *P*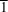 with one mol­ecule in the asymmetric unit. Fig. 6 ▸ shows a side view of its mol­ecular structure, while a top view is shown in Fig. S5.

The distance of the substituted Cp ring is nearly the same as in com­pound **4** and the staggering of the rings is also very similar. All arene rings are in axial positions, which is rather surprising, and only one is in the ‘usual’ close to perpendicular orientation with respect to the Cp ring. As in com­pound **4**, there is no observable tendency of the arene rings to orient themselves parallel to each other. The Fe⋯S distances in **6** are slightly shorter than in com­pound **4**, which parallels the observation made for com­pound **5** in comparison with com­pounds **2a** and **2b**.

### Packing plots

**Figure 7:**
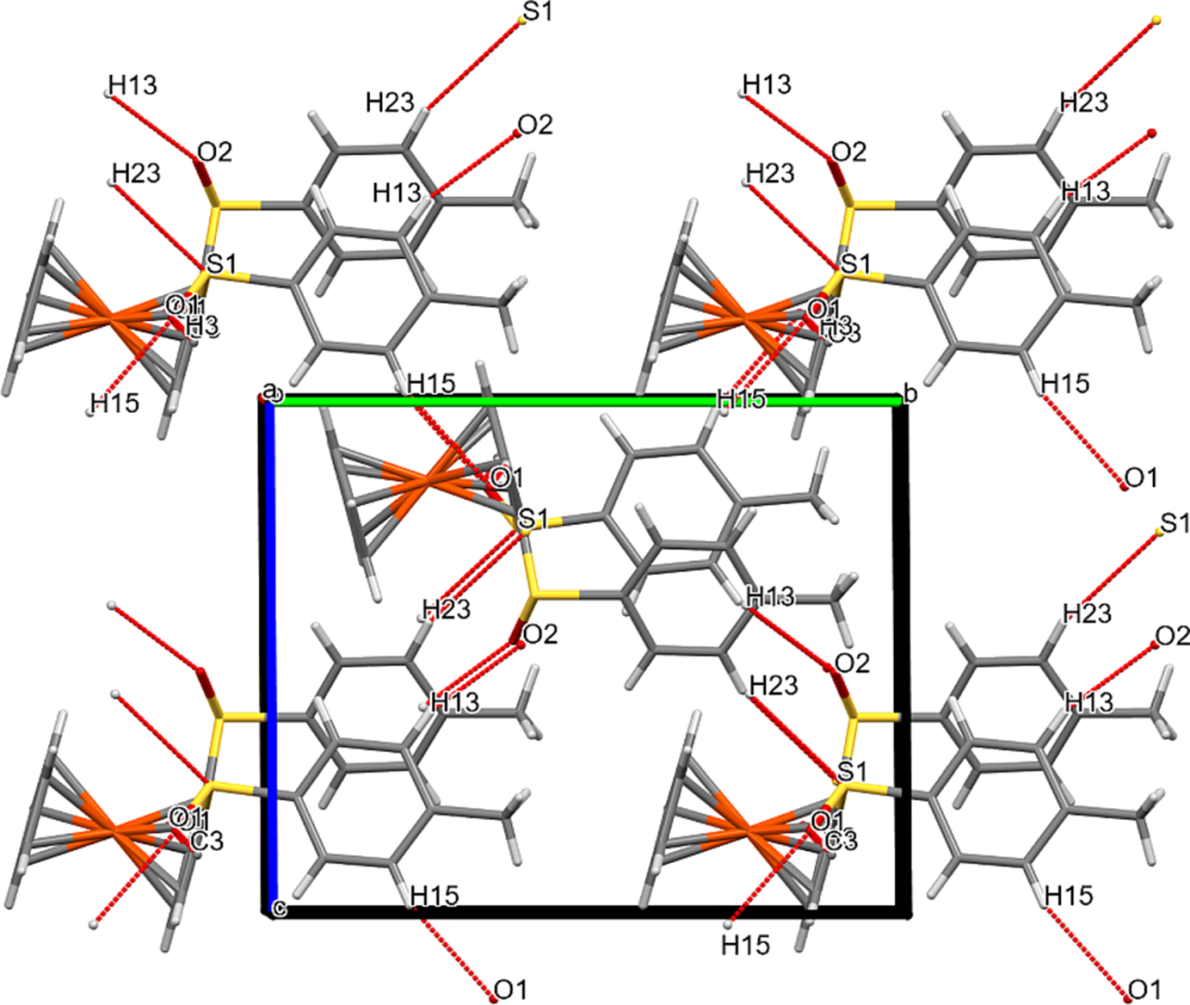
Packing plot of com­pound **2a**, viewed along *a*. The red and blue (colour coding according to the standard settings of *Mercury*: red is ‘hanging’, *i.e.* non-complete, and cyan is ‘not-hanging’, *i.e.* complete) coloured lines show weak inter­actions involving O and/or S atoms. Generic atoms labels without symmetry codes have been used.

Besides the bond parameters within a single mol­ecule, it also seemed inter­esting to look at the inter­molecular inter­actions. For this purpose, we examined the packing plots. Figs. 7 ▸–12 ▸ ▸ ▸ ▸ ▸ show the packing plots of com­pounds **2a**, **2b**, **3a**, **4**, **5** and **6**, respectively. Although there are many different ‘noncovalent inter­actions’, the plots show only the inter­molecular inter­actions that involve O or S atoms. For other types of inter­actions, see Sections 3.4 and 3.6.

In **2a**, the fer­ro­cene cores are perpendicular to the *bc* plane, while the arene rings are close to being parallel to it. A chain consisting of alternating fer­ro­cene cores and arene rings propagates in the *c* direction. O⋯*X* and S⋯*X* contacts connect the mol­ecules in all directions.

Compound **2b** shows a very different arrangement. Parallel to the *ac* diagonal run chains that contain either exclusively fer­ro­cene cores with their mol­ecular axes arranged anti­parallel to each other, or arene rings with their planes oriented nearly perpendicular to the *ac* plane. The fer­ro­cene ‘cores’ are joined in the direction of the *ac* diagonal *via* S⋯*X* and O⋯*X* inter­actions.

In com­pound **3a**, as can also be seen in Fig. S3, the fer­ro­cene axis of all the mol­ecules in the crystal are parallel to each other, and all the arene rings orient themselves perpendicular to the planes of the Cp rings. Thus, a kind of ‘mixed-layer’ structure develops. All layers are inter­connected *via* O⋯*X* and/or S⋯*X* contacts.

Figs. 10 ▸ and S6 show the packing plots for com­pound **4**. S⋯*X* and O⋯*X* contacts join the *A* mol­ecules with each other, the *B* mol­ecules with each other, as well as with *A* mol­ecules, the *A* mol­ecules with the ordered ethyl acetate solvent mol­ecules and the *B* mol­ecules with the disordered ethyl acetate solvent mol­ecules. Fig. S6 shows the ‘polymeric’ arrangement of the disordered solvent mol­ecules running at *z* = 0.5 along the *y* direction.

Compounds **5** and **6** do not contain O atoms. While **5** does not show any weak inter­actions involving the S atoms, there are several such contacts in the crystals of **6**. Inversion- and translation-related mol­ecules are thus joined in the *a* and *b* directions. As the fer­ro­cene cores are situated close to *z* = 0 and *z* = 1, the space between them is filled by arene rings. Therefore, there are no contacts involving the S atoms in the ‘long’ *c* direction.

### Hydrogen bonding: C—H⋯O, C—H⋯S and C—H⋯C contacts

For a more detailed discussion, including numerical values for these contacts, see the supporting information (chapter 2 and Tables S1–S4).

In all the sulfinyl-substituted com­pounds, all the O atoms, except for O23 in com­pound **2b**, accept hy­dro­gen bonds. Both intra- and inter­molecular C—H⋯O hy­dro­gen bonds are found, in most cases involving arene C—H bonds. The shortest H⋯O contact of 2.19 Å occurs for com­pound **3a**. The ob­served C—H⋯O angles range between 104 (intra­molecular hy­dro­gen bond) and 173° (inter­molecular hy­dro­gen bond).

S atoms rarely act as hy­dro­gen-bond acceptors for the sulfinyl com­pounds (one inter­molecular hy­dro­gen bond each in com­pounds **2a** and **3a**, and none in **2b** and **4**). Quite astonishingly, the S atoms of com­pound **5** also do not accept any hy­dro­gen bonds, while the two ‘inner’ S atoms of **6** accept one inter­molecular hy­dro­gen bond each. In the latter com­pound, the shortest H⋯S distance is 2.86 Å, while the C—H⋯S angles are in the range 143–163°.

C—H⋯C contacts of the C—H⋯π type (Mishra *et al.*, 2014 ▸) are found for all com­pounds except **2a** and **5**. There is one intra­molecular inter­action between an arene *ortho*-H atom and the attached substituted Cp ring for com­pound **3a**, and one inter­molecular inter­action between a tolyl­sul­fan­yl methyl group and an unsubstituted Cp ring for com­pound **2b**. In all other cases, the arene rings act as acceptors, mostly from other arene rings. The observed H⋯centroid distances range from 2.54 to 2.93 Å, both extrema being found in com­pound **4**.

### Chalcogen bonding: O⋯O, O⋯S and S⋯S contacts

**Figure 8:**
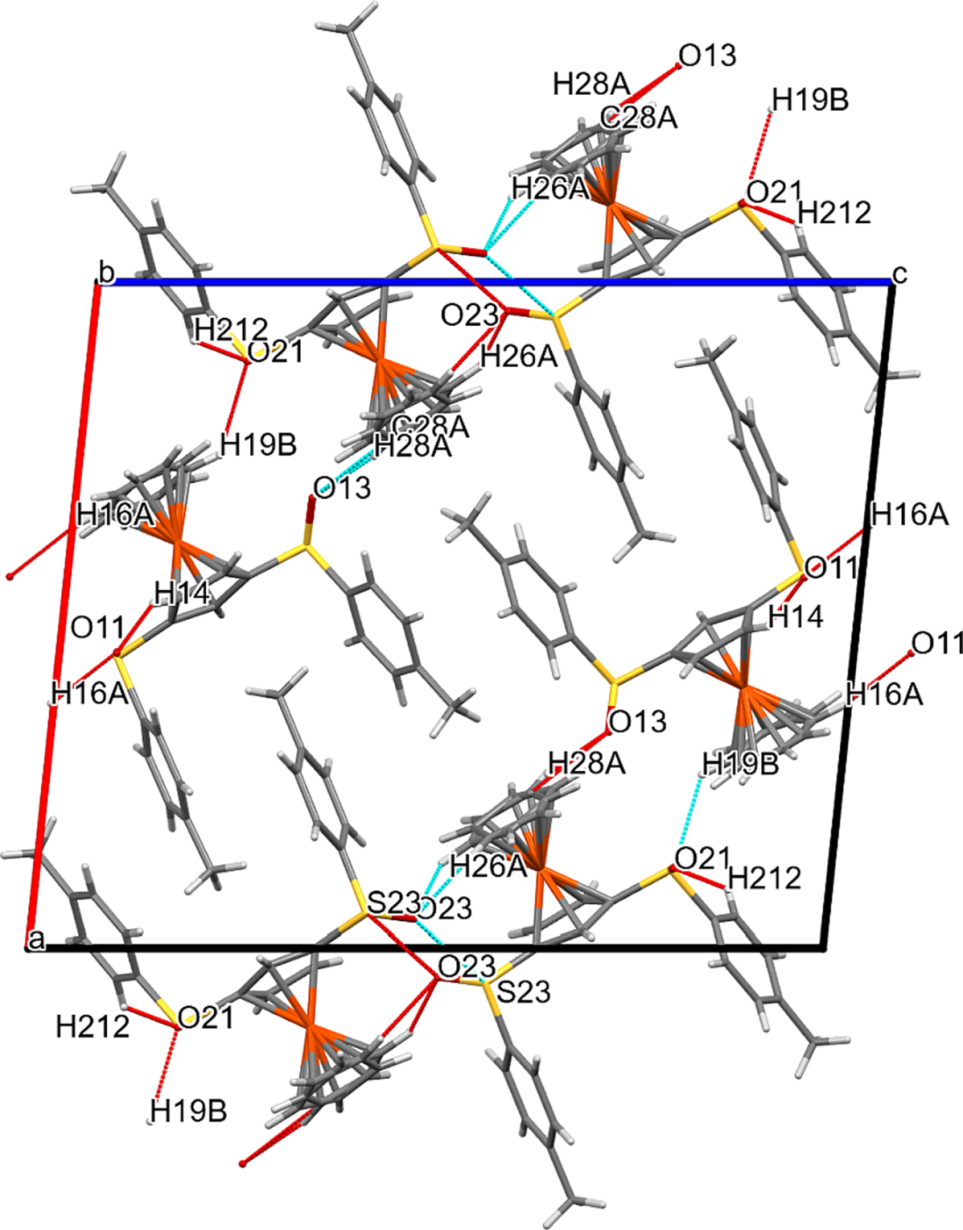
Packing plot of com­pound **2b**, viewed along *b*. The red and blue lines (for definition of colours, see Fig. 7 ▸) show weak inter­actions involving O and/or S atoms. Generic atoms labels without symmetry codes have been used.

**Figure 10:**
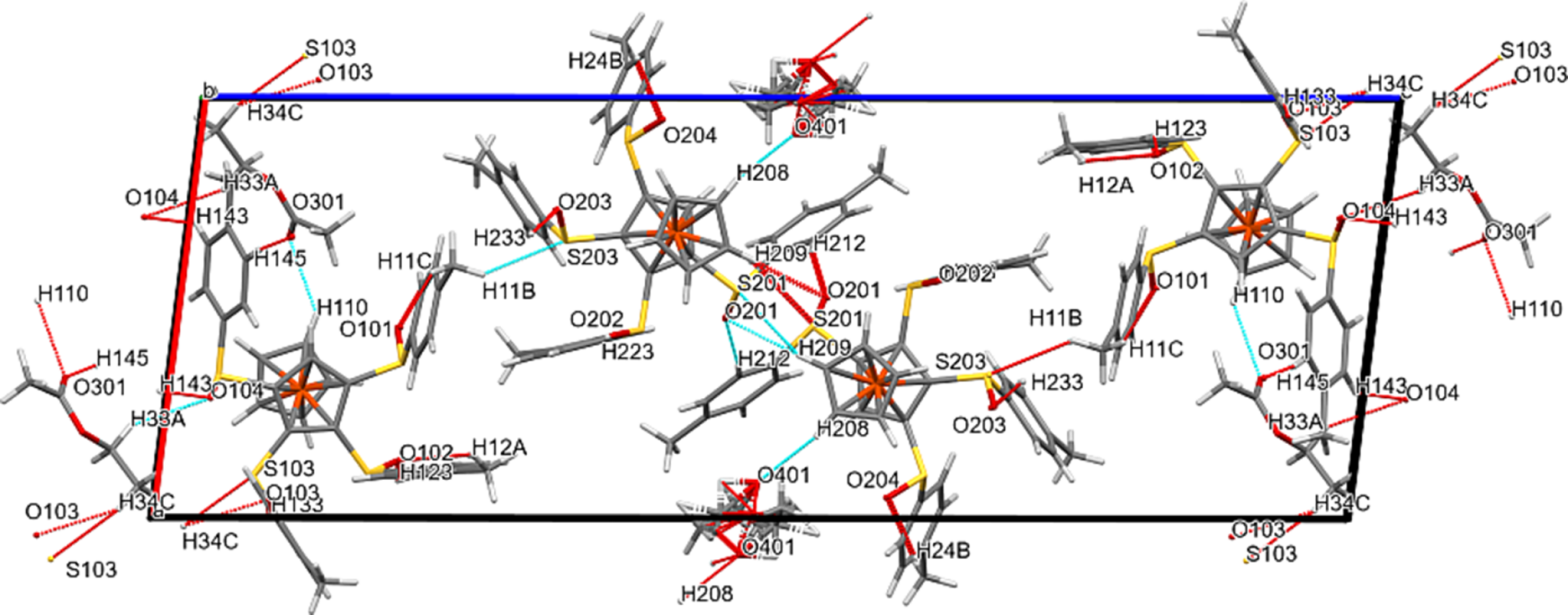
Packing plot of com­pound **4**, viewed along *b*. The red and blue coloured lines (for definition of colours, see Fig. 7 ▸) show weak inter­actions involving O or S atoms. Generic atoms labels without symmetry codes have been used.

**Figure 13:**
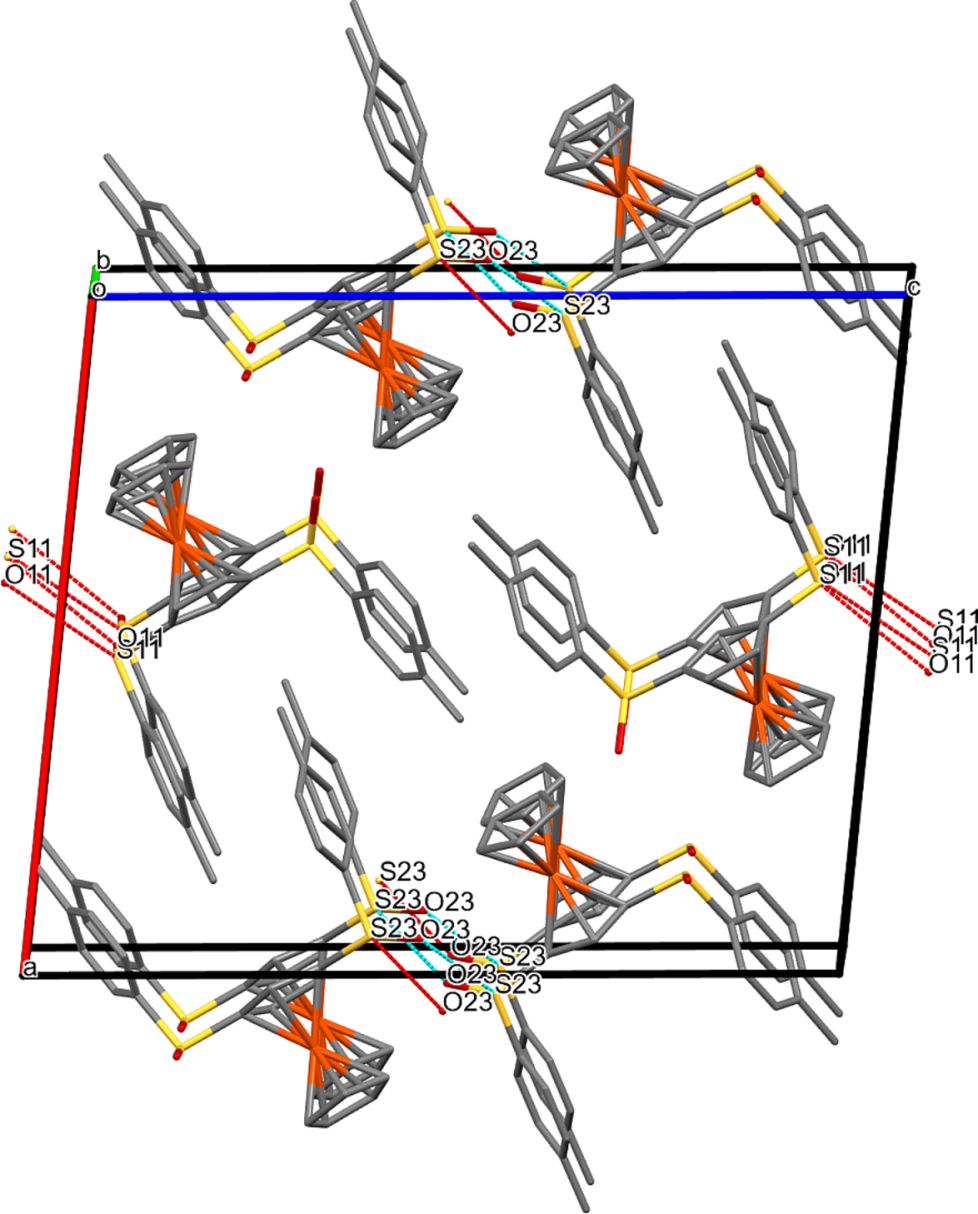
Packing plot of com­pound **2b**, viewed along *b*, showing four helices joined by S—O⋯S contacts in the *b* direction. H atoms have been omitted for clarity. Generic atoms labels without symmetry codes have been used.

We examined the structures of the six title com­pounds for the existence of chalcogen bonding, using *Mercury* (Macrae *et al.*, 2020 ▸); however, only **2b** showed such inter­actions. In this com­pound, the mol­ecules are joined into a helix along the crystallographic 2_1_ screw axis *via* an S23⋯O23 inter­action. The inter­molecular S⋯O distance is 3.278 (4) Å, with an S—O⋯S angle of 152.4 (2)° and an O—S⋯O angle of 82.5 (2)°. Another much weaker S⋯O inter­action, supporting this helical arrangement, involves S11 and O11; the corresponding parameters are S⋯O = 3.779 (4) Å, S—O⋯S = 117.0 (2)° and O—S⋯O = 110.9 (2)°. These inter­actions can been seen in Fig. 8 ▸; however, for a clearer understanding, Fig. 13 ▸ shows these inter­actions more explicitly.

### C⋯C contacts and short ring–ring inter­actions

This analysis was performed using *PLATON* (Spek, 2020 ▸). There are many ‘short’ distances between ring centroids below the *PLATON* limit of 6.0 Å, which might indicate some kind of π–π inter­actions. We restrict the present discussion to such inter­actions below the 4.5 Å distance limit. There are no such inter­actions for com­pounds **2b**, **4**, **5** and **6**.

**Figure 14:**
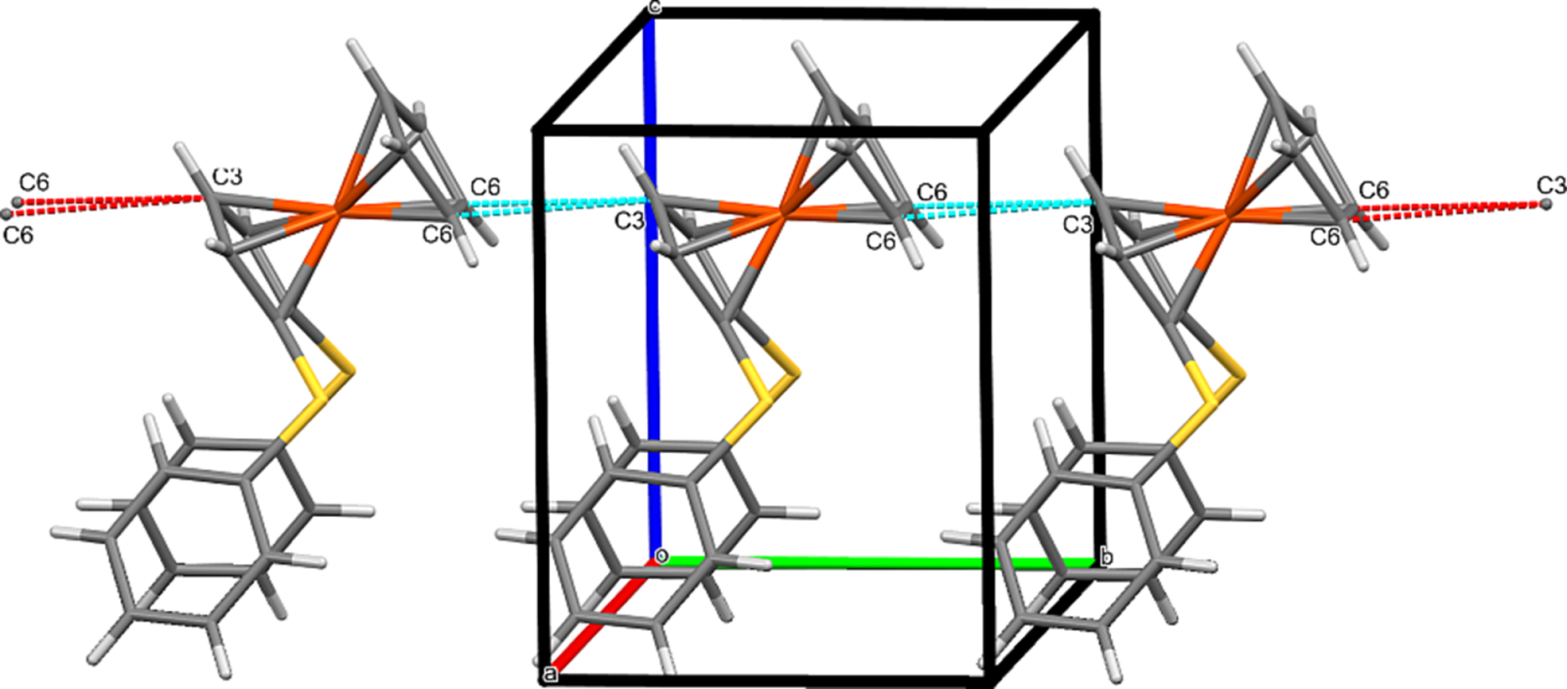
The polymeric arrangement of fer­ro­cene cores in com­pound **5**. Generic atoms labels without symmetry codes have been used.

In com­pound **2a**, there is a rather long intra­molecular inter­action of 4.156 (3) Å between the ring centroids. Quite inter­estingly, in corresponding disul­fan­yl com­pound **5**, the distance between the centroids is much longer at 4.716 (3) Å. However, there is an inter­esting C⋯C inter­action in the latter com­pound between a Cp C atom of the substituted ring with two Cp atoms of the unsubstituted ring of the next mol­ecule in the *b* direction, producing a ‘polymeric’ arrangement (Fig. 14 ▸).

In com­pound **3a**, there are close intra­molecular contacts between arene rings within mol­ecule *A* and within mol­ecule *B*, as well as close inter­molecular contacts between mol­ecules *A* and *B* (Table S5). Most distances between centroids range from 3.70 to 3.90 Å, with only one long distance of *ca* 4.30 Å. Fig. S7 shows these inter­actions and a closer inspection shows that some C atoms still do not take part, with mol­ecule *B* having a higher number of such ‘unbound’ C atoms.

### Hirshfeld analysis

In order to gain further insight into the inter­molecular inter­actions, we performed a Hirshfeld analysis using the program *CrystalExplorer* (Spackman *et al.*, 2021 ▸), which allows not only the calculation of the Hirshfeld surfaces, but also of so-called ‘fingerprint plots’ (Spackman & McKinnon, 2002 ▸) and ‘inter­action energies’ (Spackman, 2015 ▸; Mackenzie *et al.*, 2017 ▸).

#### Fingerprint plots

Analysis of the fingerprint plots allows the relative contributions of element-pair inter­actions across the Hirshfeld surface to be determined (Figs. S8–S10 and Table 4 ▸). Within the graphical representations, grey areas represent the absence of any close inter­actions, while dark-blue and light-blue areas represent an increasing number of inter­actions. A first quick look at Fig. S8 shows that several plots of mol­ecules *A* and *B* of com­pound **2b** look quite different from each other, and also different from the plots of the stereoisomeric **2a**, and particularly different from sul­fan­yl com­pound **5**. For example, the closest H⋯H contacts in **2a** occur at *d*_i_ + *d*_e_ = 2.15 Å, in mol­ecule *A* of **2b** at *ca* 2.10 Å, while in mol­ecule *B* they are at *ca* 2.00 Å and in com­pound **5** at 2.5 Å. Similar differences occur between the two mol­ecules of com­pounds **4** and **6** (Fig. S10). While there are some subtle differences between the two mol­ecules of com­pound **3a**, they are not as obvious as in the other com­pounds (Fig. S9).

As can be seen from Table 4 ▸, for all com­pounds, the major inter­actions are of the H⋯H type, followed by C⋯H inter­actions, except for com­pound **3a**, where O⋯H inter­actions are the second most common. It is astonishing that S⋯S inter­actions are not significant for any com­pound, and O⋯O and S⋯O inter­actions provide only small contributions in com­pound **2b**, while they are not significant for the other com­pounds. By far the largest contribution comes from H⋯H inter­actions in both mol­ecules of com­pound **3a**, making up for nearly two thirds of all contributions. The largest values for the C⋯H and S⋯H contributions are found for polysul­fan­yl com­pounds **5** and **6**, where they make up for nearly one-third and approximately one-ninth, respectively. There are no recognizable trends with respect to the degree of substitution.

#### Inter­action energies

Inter­action energies were calculated using the program *CrystalExplorer*, using *TONTO* at the HF/3-21G level. For the discussion, only contributions with |*E*_tot_| > 10 kJ mol^−1^ were used, usually between six and nine contributors (see Tables S6–S8 in the supporting information). For all com­pounds, the dispersion term was the most important, and in only a few cases was the electronic term of similar importance. When comparing only the strongest inter­actions (Type A) of all com­pounds, the following ‘ranking’ of the |*E*_tot_| values results: **4** > **2b** ≃ **6** > **3a** > **2a** > **5**. When considering only the electronic terms, there are only five contributors with |*E*_ele_| > 25 kJ mol^−1^, with a ranking of **4** (types A and B) > **3a** (types B and A) > **2b** (type C). There is a general trend of increasing inter­action energies with increasing degree of substitution, with the surprising exception of 1,3-disubstituted com­pound **2b**, and stronger inter­actions for the sulfinyl com­pounds compared with the sul­fan­yl com­pounds with the same degree of substitution.

It is quite difficult to compare these results with the literature data, as hardly any Hirshfeld analysis data with respect to inter­action energies have been reported, either for metallocenes or for sul­fan­yl or sulfinyl com­pounds. For the former, only 1,1′-di­methyl­fer­ro­cene is reported (Mackenzie *et al.*, 2017 ▸), while for the latter, only the above-mentioned article by Zhou *et al.* (2021 ▸) applies. In both cases, the observed inter­action energies were much lower than the maximal values found here {|*E*_ele_| < 9 kJ mol^−1^ for [Fe(C_5_H_4_Me)_2_]}. However, we found a series of structurally related aromatic thio­ethers of the type [C_6_(SPh)_4_(CN)_2_] in the Cambridge Structural Database (CSD; Groom *et al.*, 2016 ▸), and chose one of them (VOHFOR; Schmiedtchen *et al.*, 2023 ▸) for examination by *CrystalExplorer*. And, indeed, it turned out that similar high inter­action energies were calculated with this com­pound (*E*_ele_ ≃ −40 kJ mol^−1^ and *E*_tot_ ≃ −70 kJ mol^−1^; Table S9). A detailed comparison of the latter structure with com­pound **4** can be found in the supporting information. It seems therefore most likely that the large number of inter­acting –SPh groups is responsible for the observed large inter­action energies.

## Conclusion

We have shown that even with only a slight excess of LDA, com­pound **1** undergoes reactions that involve poly­func­tion­al­ization of the Cp ring. Chromatography allows isolation of pure disubstituted (1,2- and 1,3-isomers), tris­ubstituted (1,2,3-isomer) and tetra­substituted products.

The mol­ecular structures of all the com­pounds show a relatively small influence of the degree of substitution on the typical metallocene bond parameters (Fe–centroid distances and relative orientations of the Cp rings), while there seems to be no difference between the corresponding sulfinyl and sul­fan­yl com­pounds. In all the sulfinyl­fer­ro­cenes, the O atoms are in equatorial positions, except for one O atom in com­pound **4**, while all the arene rings (again except for one) orient themselves perpendicular to the plane of the Cp ring.

Many C—H⋯O hy­dro­gen bonds are observed in all of the sulfinyl com­pounds, while C—H⋯S contacts are seen rarely in compounds **2a**, **2b**, **3a** and **4**, while they gain some im­portance for tetra­sul­fan­ylfer­ro­cene **6**. C—H⋯π inter­actions occur for all the com­pounds except the 1,2-disubstituted ones. Chalcogen bonding is seen only in 1,3-di­substituted sul­fin­yl­fer­ro­cene **2b**. Significant π–ring inter­actions are only observed for com­pound **3a** and are mainly intra­molecular.

Hirshfeld analysis shows that H⋯H and C⋯H inter­actions are the most important, except for tris­ulfinylfer­ro­cene **3a**, where H⋯H and H⋯O inter­actions are of the highest importance. Calculation of the inter­action energies shows that for all com­pounds the dispersion terms are the most important. A ‘ranking’ of the total energies shows a general trend of increasing inter­action energies (absolute values) with increasing degree of substitution, and with higher values for sulfinyl than for sul­fan­yl com­pounds.

Although the optical purity was not checked for any of the com­pounds, the values for the Flack parameters in the structures of **2a**, **2b**, **3a** and **4** suggest that the observed stereochemical outcome of the reactions resembles the expectation, *i.e.* always *S*_S_. Therefore, it seems to us worthwhile to study the outcome of reactions when using either the opposite enanti­omer or racemates.

## Supplementary Material

Crystal structure: contains datablock(s) comp_2a, compd_2b, comp_3a, comp_4, comp_5, comp_6, global. DOI: 10.1107/S2053229624009318/ux3008sup1.cif

Structure factors: contains datablock(s) comp_2a. DOI: 10.1107/S2053229624009318/ux3008comp_2asup2.hkl

Structure factors: contains datablock(s) compd_2b. DOI: 10.1107/S2053229624009318/ux3008compd_2bsup3.hkl

Structure factors: contains datablock(s) comp_3a. DOI: 10.1107/S2053229624009318/ux3008comp_3asup4.hkl

Structure factors: contains datablock(s) comp_4. DOI: 10.1107/S2053229624009318/ux3008comp_4sup5.hkl

Structure factors: contains datablock(s) comp_5. DOI: 10.1107/S2053229624009318/ux3008comp_5sup6.hkl

Structure factors: contains datablock(s) comp_6. DOI: 10.1107/S2053229624009318/ux3008comp_6sup7.hkl

Experimental details, hydrogen-bonding interactions, interaction energies and figures. DOI: 10.1107/S2053229624009318/ux3008sup8.pdf

CCDC references: 2385978, 2385977, 2330913, 2330911, 2330912, 2330910

## Figures and Tables

**Figure 9:**
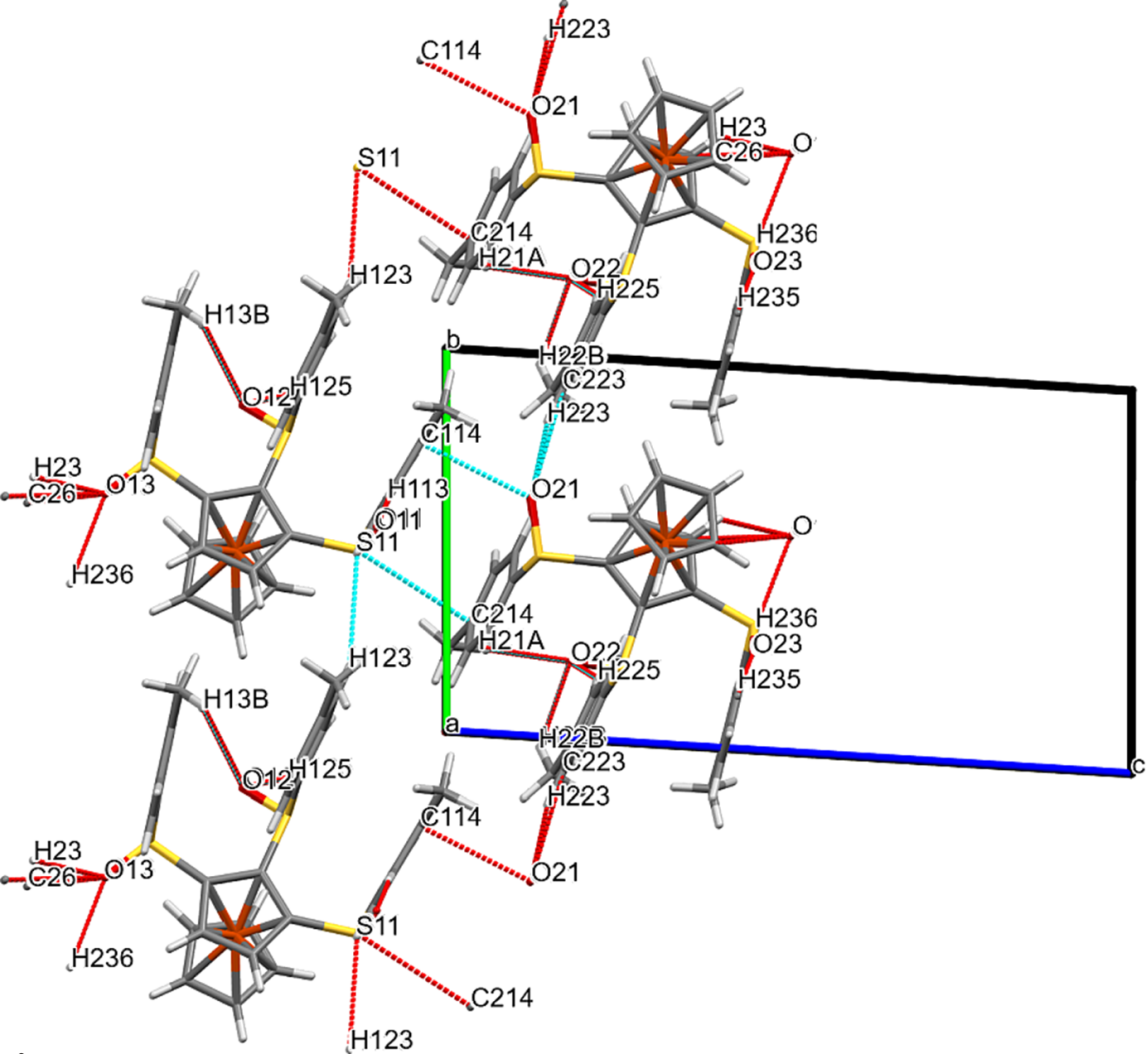
Packing plot of com­pound **3a**, viewed perpendicular to the Cp ring planes. The red and blue coloured lines (for definition of colours, see Fig. 7 ▸) show weak inter­actions involving O and/or S atoms. Generic atoms labels without symmetry codes have been used.

**Figure 11:**
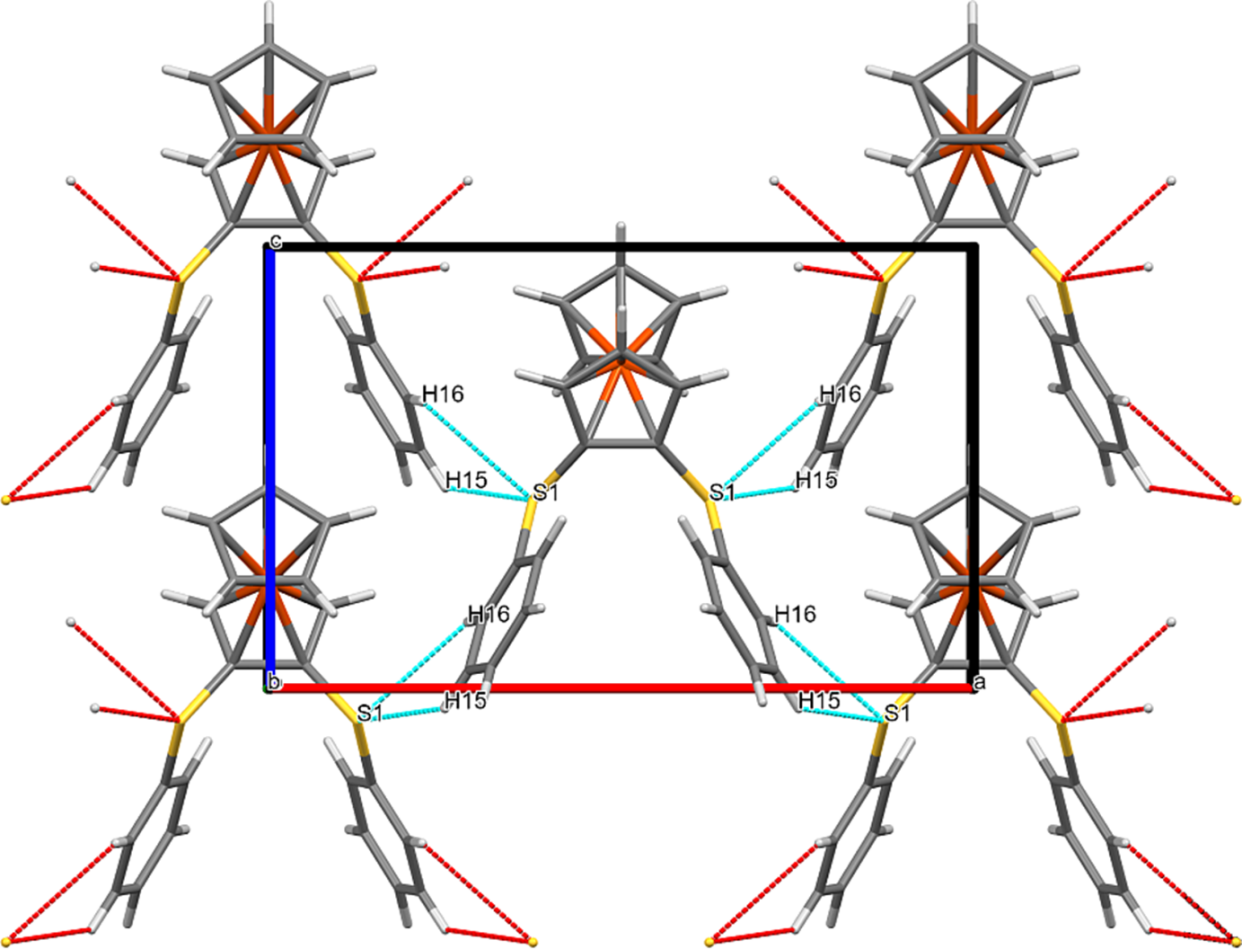
Packing plot of com­pound **5**, viewed along the *b* axis. Generic atoms labels without symmetry codes have been used.

**Figure 12:**
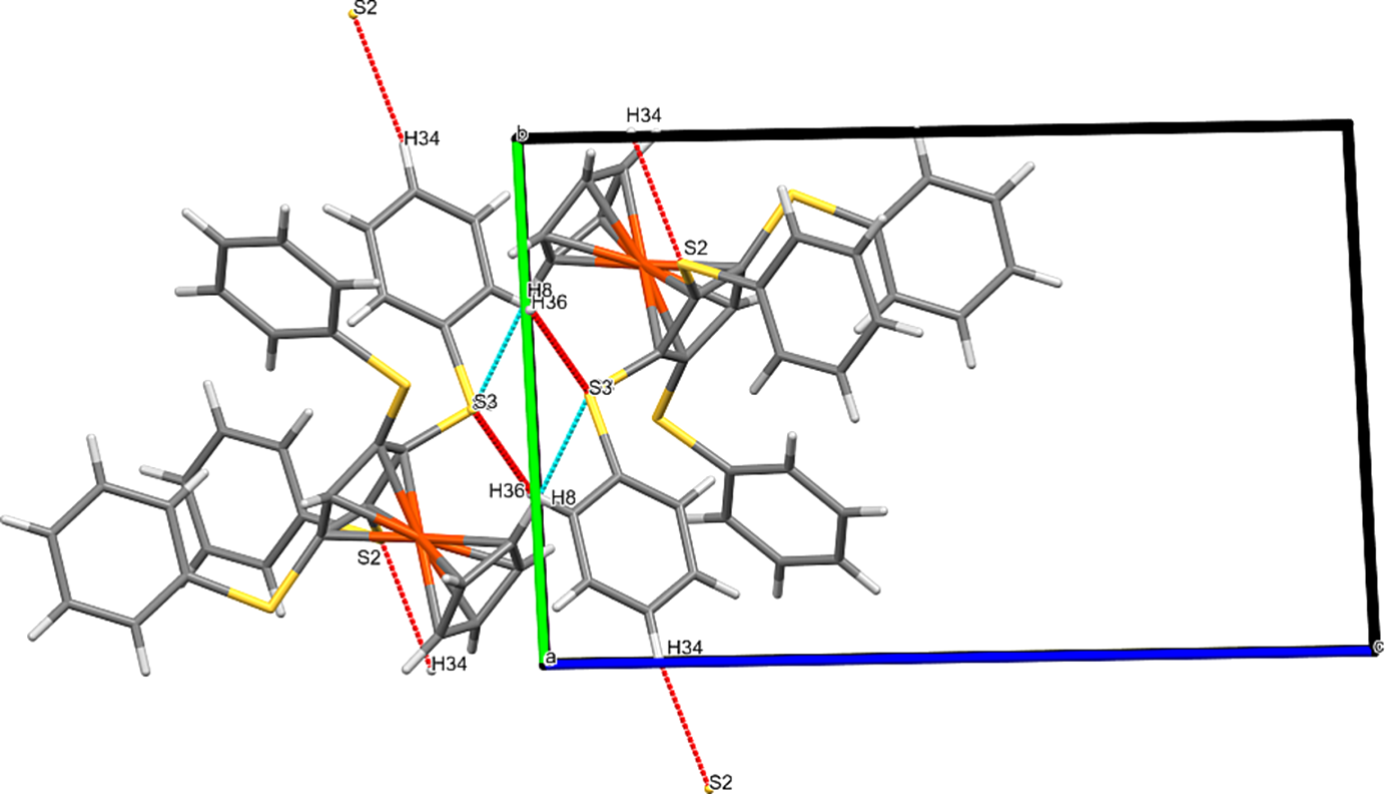
Packing plot of com­pound **6**, viewed along *a*. The red and blue coloured lines (for definition of colours, see Fig. 7 ▸) show weak inter­actions involving S atoms. Generic atoms labels without symmetry codes have been used.

**Table:** Experiments were carried out with Mo *K*α radiation using a Bruker D8 VENTURE diffractometer. Absorption was corrected for by multi-scan methods (*SADABS*; Krause *et al.*, 2015 ▸). H-atom parameters were constrained.

	**2a**	**2b**	**3a**
Crystal data			
Chemical formula	[Fe(C_5_H_5_)(C_19_H_17_O_2_S_2_)]	[Fe(C_5_H_5_)(C_19_H_17_O_2_S_2_)]	[Fe(C_5_H_5_)(C_26_H_23_O_3_S_3_)]
*M*_r_	462.38	462.38	600.56
Crystal system, space group	Monoclinic, *P*2_1_	Monoclinic, *P*2_1_	Triclinic, *P*1
Temperature (K)	297	296	110
*a*, *b*, *c* (Å)	7.8964 (2), 12.9064 (3), 11.0124 (3)	17.1882 (10), 6.0383 (4), 20.4271 (12)	7.8298 (5), 9.8573 (6), 17.4937 (11)
α, β, γ (°)	90, 109.467 (1), 90	90, 95.995 (2), 90	93.379 (2), 91.120 (2), 98.051 (2)
*V* (Å^3^)	1058.16 (5)	2108.5 (2)	1334.02 (14)
*Z*	2	4	2
μ (mm^−1^)	0.93	0.93	0.83
Crystal size (mm)	0.07 × 0.05 × 0.04	0.10 × 0.02 × 0.02	0.10 × 0.03 × 0.02

Data collection			
*T*_min_, *T*_max_	0.679, 0.746	0.648, 0.746	0.809, 0.862
No. of measured, independent and observed [*I* > 2σ(*I*)] reflections	11282, 4815, 4393	34842, 10422, 8415	21012, 10957, 9889
*R*_int_	0.020	0.032	0.030
(sin θ/λ)_max_ (Å^−1^)	0.649	0.667	0.634

Refinement			
*R*[*F*^2^ > 2σ(*F*^2^)], *wR*(*F*^2^), *S*	0.034, 0.084, 1.14	0.042, 0.095, 1.02	0.034, 0.073, 1.02
No. of reflections	4815	10422	10957
No. of parameters	264	573	691
No. of restraints	13	8	6
Δρ_max_, Δρ_min_ (e Å^−3^)	0.41, −0.34	0.50, −0.23	0.41, −0.30
Absolute structure	Flack *x* determined using 1876 quotients [(*I*^+^) − (*I*^−^)]/[(*I*^+^) + (*I*^−^)] (Parsons *et al.*, 2013 ▸)	Flack *x* determined using 3063 quotients [(*I*^+^) − (*I*^−^)]/[(*I*^+^) + (*I*^−^)] (Parsons *et al.*, 2013 ▸)	Flack *x* determined using 4237 quotients [(*I*^+^) − (*I*^−^)]/[(*I*^+^) + (*I*^−^)] (Parsons *et al.*, 2013 ▸)
Absolute structure parameter	0.014 (6)	0.019 (7)	0.014 (8)

**Table:** 

	**4**	**5**	**6**
Crystal data			
Chemical formula	[Fe(C_5_H_5_)(C_33_H_29_O_4_S_4_)]·0.75C_4_H_8_O_2_	[Fe(C_5_H_5_)(C_17_H_13_S_2_)]	[Fe(C_5_H_5_)(C_29_H_21_S_4_)]
*M*_r_	804.82	402.33	618.64
Crystal system, space group	Monoclinic, *P*2_1_	Orthorhombic, *Pmn*2_1_	Triclinic, *P* 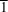
Temperature (K)	107	110	110
*a*, *b*, *c* (Å)	12.8893 (7), 8.2225 (4), 36.500 (2)	14.0977 (11), 7.1607 (5), 8.8504 (5)	8.4836 (4), 10.3028 (5), 16.7210 (8)
α, β, γ (°)	90, 97.106 (2), 90	90, 90, 90	90.730 (2), 103.948 (2), 94.999 (2)
*V* (Å^3^)	3838.6 (4)	893.44 (11)	1412.15 (12)
*Z*	4	2	2
μ (mm^−1^)	0.66	1.08	0.85
Crystal size (mm)	0.07 × 0.02 × 0.02	0.10 × 0.08 × 0.05	0.06 × 0.05 × 0.02

Data collection			
*T*_min_, *T*_max_	0.811, 0.862	0.682, 0.746	0.691, 0.746
No. of measured, independent and observed [*I* > 2σ(*I*)] reflections	59999, 15634, 13598	9831, 2561, 2426	23494, 7023, 5898
*R*_int_	0.074	0.041	0.046
(sin θ/λ)_max_ (Å^−1^)	0.625	0.694	0.667

Refinement			
*R*[*F*^2^ > 2σ(*F*^2^)], *wR*(*F*^2^), *S*	0.065, 0.141, 1.12	0.025, 0.060, 1.05	0.034, 0.088, 1.04
No. of reflections	15634	2561	7023
No. of parameters	936	118	352
No. of restraints	77	1	0
Δρ_max_, Δρ_min_ (e Å^−3^)	0.69, −0.56	0.30, −0.31	0.42, −0.39
Absolute structure	Flack *x* determined using 4943 quotients [(*I*^+^) − (*I*^−^)]/[(*I*^+^) + (*I*^−^)] (Parsons *et al.*, 2013 ▸)	Flack *x* determined using 1069 quotients [(*I*^+^) − (*I*^−^)]/[(*I*^+^) + (*I*^−^)] (Parsons *et al.*, 2013 ▸)	–
Absolute structure parameter	0.053 (8)	0.018 (9)	–

Computer programs: *APEX2* (Bruker, 2011 ▸), *SAINT* (Bruker, 2011 ▸), *SHELXT2014*/*SHELXT2018* (Sheldrick, 2015*a* ▸), *SHELXL2018*/*SHELXL2019* (Sheldrick, 2015*b* ▸), *ORTEP-3 for Windows* (Farrugia, 2012 ▸), *Mercury* (Macrae *et al.*, 2020 ▸) and *PLATON* (Spek, 2020 ▸).

**Table 2:** Important bond parameters (Å, °) for **2a**, **2b** and **5** in comparison with some related com­pounds from the literature CT is the centroid of a Cp ring, the subscript ‘sub’ refers to the substituted Cp ring, the subscript ‘C5H5’ refers to the unsubstituted Cp ring, C_i_ is an arene *ipso*-C atom and C_o_ is an arene *ortho*-C atom.

Distances/angles	**2a**	**2b** (molecule *A*)	**2b** (molecule *B*)	**1** (VEZPUM)	HEZMIJ	TELHOH	**5**
Fe—CT_sub_	1.629 (2)	1.634 (2)	1.631 (2)	1.647/1.643	1,647/1.641	1.634 (3)	1.6305 (11)
Fe—CT_C5H5_	1.653 (2)	1.644 (4)	1.660 (4)	1.657/1.656	1.654/1.650	1.650 (3)	1.6512 (13)
C_sub_—CT_sub_—CT_C5H5_—C_C5H5_	5.2	14.0	9.9	0.2/1.7	15.2/10.6	6.4 (5)	0.2
C_sub_—S	1.770 (3)	1.779 (4)	1.778 (4)	1.779 (2)/	1.770 (3)/	1.774 (6)	1.752 (2)
	1.757 (3)	1.764 (4)	1.779 (4)	1.777 (2)	1.768 (3)		
S—O	1.478 (4)	1.490 (4)	1.479 (5)	1.496 (2)/	1.498 (2)/	1.502 (5)	
	1.498 (4)	1.476 (4)	1.488 (4)	1.496 (1)	1.491 (2)		
Fe⋯O	3.694 (3)	3.777 (3)	3.772 (4)	3.803 (2)/	4.589 (1)/	4.555 (5)	
	3.725 (3)	3.686 (4)	3.822 (3)	3.782 (2)	4.581 (1)		
Fe⋯S	3.333 (1)	3.365 (1)	3.340 (1)	3.3634 (8)	3.4042 (6)	3.409 (2)	3.3038 (7)
	3.288 (1)	3.334 (1)	3.345 (1)	3.3180 (8)	3.4062 (6)		
C_sub_—S—O	103.4 (2)	105.4 (6)	105.7 (2)	107.1 (1)/	107.9 (2)/	105.6 (3)	
	108.6 (2)	106.7 (3)	107.0 (2)	106.9 (1)	106.5 (1)		
CT_C5H5_—CT_sub_—S—O	73.6	61.4	73.5	66.3/	172.4/	170.1	
	62.9	66.3	71.4	74.0	176.1		
O—S—C_i_—C_o_	37.8 (4)	14.3 (4)	28.8 (4)	21.3 (2)/	21.1 (2)/	14.0 (6)	
	27.7 (4)	12.4 (6)	32.2 (5)	19,7(2)	24.9 (2)		
O—S—C_sub_—C	14.9 (3)	20.6 (4)	12.8 (4)	23.6 (3)/	86.0 (2)/	79.6 (3)	
	23.2 (3)	27.8 (5)	14.9 (5)	13.2 (2)	82.7 (2)		
C_sub_—S—C_i_—C_o_	44.0 (4)	84.9 (5)	82.8 (4)	85.7 (2)/	91.1 (1)/	90.4 (6)	7.0 (2)
	69.4 (4)	59.3 (4)	72.5 (4)	88.9 (2)	89.6 (1)		
∠(Cp, Ph)	80.9 (2)	85.6 (3)	81.6 (2)	82.6/	86.4/	84.7	89.63 (12)
	86.4 (2)	86.3 (3)	81.4 (3)	84.0	78.5		
∠(Ph, Ph)	32.3 (3)	17.2 (2)	37.6 (2)				60.59 (6)

**Table 3:** Important bond parameters (Å, °) in com­pounds **3a**, **4** and **6** CT is the centroid of a Cp ring, the subscript ‘sub’ refers to the substituted Cp ring, the subscript ‘C5H5’ refers to the unsubstituted Cp ring, C_i_ is an arene *ipso*-C atom and C_o_ is an arene *ortho*-C atom.

Distances/angles	**3a** (molecule *A*)	**3a** (molecule *B*)	**4** (molecule *A*)	**4** (molecule *B*)	**6**
Fe—CT_sub_	1.629 (2)	1.623 (2)	1.621 (3)	1.622 (3)	1.6196 (8)
Fe—CT_C5H5_	1.653 (2)	1.661 (3)	1.664 (4)	1.660 (4)	1.6668 (11)
C_sub_—CT_sub_—CT_C5H5_—C_C5H5_	2.5	0.88	20.9	8.6	18.4
C_sub_—S	1.779 (4)	1.788 (4)	1.791 (7)	1.784 (8)	1.756 (2)
	1.773 (4)	1.776 (4)	1.786 (7)	1.795 (8)	1.753 (2)
	1.791 (4)	1.791 (4)	1.793 (7)	1.801 (8)	1.754 (2)
			1.786 (8)	1.801 (8)	1.756 (2)
S—O	1.486 (3)	1.492 (3)	1.486 (6)	1.488 (6)	
	1.496 (3)	1.495 (3)	1.480 (6)	1.490 (6)	
	1.494 (3)	1.486 (3)	1.485 (6)	1.480 (6)	
			1.490 (6)	1.490 (7)	
Fe⋯O	3.856 (3)	3.850 (3)	4.518 (6)	4.455 (6)	
	3.800 (3)	3.698 (3)	3.550 (5)	3.643 (6)	
	3.613 (2)	3.593 (3)	3.671 (6)	3.665 (6)	
			3.480 (6)	3.470 (7)	
Fe⋯S	3.370 (1)	3.319 (1)	3.407 (2)	3.302 (2)	3.3339 (6)
	3.386 (1)	3.441 (1)	3.305 (2)	3.404 (2)	3.3336 (6)
	3.337 (1)	3.324 (1)	3.400 (2)	3.341 (2)	3.2381 (5)
			3.356 (2)	3.340 (2)	3.3717 (6)
C_sub_—S—O	107.9 (2)	103.6 (2)	107.4 (3)	104.8 (3)	,
	105.9 (2)	105.9 (2)	108.8 (3)	108.2 (3)	
	103.0 (2)	108.5 (2)	107.6 (3)	108.8 (4)	
			104.7 (3)	107.2 (4)	
CT_C5H5_—CT_sub_—S—O	84.5	72.1	34.9	42.7	
	69.2	67.2	44.0	44.3	
	49.8	49.0	46.8	51.6	
			156.4	152.1	
O—S—C_i_—C_o_	3.1 (5)	34.8 (5)	12.2 (7)	31.8 (7)	
	74.0 (4)	66.5 (3)	12.0 (7)	1.5 (7)	
	40.7 (4)	5.4 (4)	2.5 (7)	8.0 (7)	
			35.1 (9)	22.8 (8)	
O—S—C_sub_—C	36.4 (4)	14.2 (4)	53.6 (8)	67.3 (7)	
	20.9 (4)	25.8 (4)	47.5 (7)	35.9 (8)	
	4.4 (4)	37.2 (4)	36.7 (7)	44.7 (8)	
			70.7 (7)	41.4 (8)	
C_sub_—S—C_i_—C_o_	65.1 (4)	71.6 (4)	66.0 (7)	53.8 (8)	8.1 (2)
	1.3 (5)	5.0 (4)	61.3 (6)	64.3 (7)	15.8 (2)
	74.2 (4)	74.4 (4)	72.2 (7)	66.6 (6)	57.2 (2)
			73.3 (7)	77.0 (7)	13.7 (2)
∠(Cp,Ph)	84.5 (2)	81.6 (2)	87.9 (4)	80.6 (4)	67.27 (11)
	89.8 (2)	81.4 (2)	81.8 (4)	88.6 (4)	87.37 (10)
	87.4 (2)	86.0 (2)	85.6 (4)	86.1 (4)	67.32 (10)
			73.0 (4)	86.1 (4)	71.45 (10)
∠(Ph,Ph)	6.4 (2)	6.4 (2)	71.4 (4)	22.7 (4)	42.81 (11)
	18.6 (2)	15.9 (2)	46.8 (4)	88.5 (4)	25.13 (10)
	14.1 (2)	12.8 (2)	22.2 (4)	33.3 (4)	58.66 (10)
			62.8 (4)	69.2 (4)	66.05 (10)
			85.9 (4)	52.7 (4)	34.45 (10)
			40.8 (4)	56.5 (4)	83.19 (9)

**Table 4:** Percentages of the individual contributions to the inter­actions across the Hirshfeld surface

	C⋯C	H⋯H	O⋯O	S⋯S	S⋯O	C⋯H	O⋯H	S⋯H
**2a**	1.5	59.6	0	0	0	19.6	13.7	5.6
**2b** (molecule *A*)	0.2	57.0	0	0	0.5	26.8	10.3	5.3
**2b** (molecule *B*)	0.2	54.9	0.3	0	0.8	27.2	13.5	3.5
**3a** (molecule *A*)	2.9	65.5	0	0	0	9.5	14.6	5.6
**3a** (molecule *B*)	2.9	64.5	0	0	0	8.5	16.8	5.3
**4**	0.7	58.5	0	0	0	19.1	15.5	4.6
**5**	0.0	57.2	–	0	–	32.4	–	10.4
**6**	0.0	52.7	–	0.9	–	31.9	–	13.1
