# Supplementary material for: Mol­ecular and crystal structures of six poly(arylsulfin­yl)- and poly(aryl­sulfan­yl)fer­ro­cenes
Source: Acta Crystallogr C Struct Chem. 2024 Oct 4;80(Pt 11):716–27. doi: 10.1107/S2053229624009318 (PMC11535883; doi:10.1107/S2053229624009318)
Supplement: Supplementary file 8 [file c-80-00716-sup8.pdf]

Supporting Information

Table of Contents

1. EXPERIMENTAL SECTION (more detailed description) 3
2. HYDROGEN BONDING AND Π-INTERACTIONS 4
3. INTERACTION ENERGIES 8
4. FIGURES 12

Figure S 1. Molecular structure of compound **2a**, top view. 12
Figure S 2 Molecular structure of compound **3a**, top views of molecules A and B 12
Figure S 3. Molecular structure of compound **3a**, molecules A and B. Top view. 13
Figure S 4 Molecular structure of compound **4**, top views of molecules A and B 13
Figure S 5. MOLECULAR STRUCTURE of compound **6**: top view 14
Figure S 6. MERCURY packing plot of compound **4** 15
Figure S 7 C…C interactions between the phenyl rings of compound **3a..** 15
Figure S 8 Individual Fingerprint plots of the disubstituted compounds **2a**, **2b** and **5** 16
Figure S 9 Individual Fingerprint plots of the tri-substituted compound **3a** 16
Figure S 10 Individual Fingerprint plots of the tetrasubstituted compounds **4** and **6**. 17
Figure S 11 Individual Contributions A-F to the Interaction Energies of compound **2a** 17
Figure S 12 Individual Contributions A-I to the Interaction Energies of compound **2b** 18
Figure S 13 Individual Contributions A-H to the Interaction Energies of compound **3a** 18
Figure S 14 Individual Contributions A-I to the Interaction Energies of compound **4** 19
Figure S 15 Individual Contributions A-G to the Interaction Energies of compound **5** 19
Figure S 16 Individual Contributions A-I to the Interaction Energies of compound **6** 20
Figure S 17: Interaction Energies for Compound **4**: the two strongest interactions 21
Figure S 18 Interaction Energies for literature Compound **VOHFOR**: the two strongest interactions. 22
Figure S 19. ^{1}H NMR spectrum (270 MHz, $CDCl_3$) of compound **1** 23
Figure S 20. ^{1}H NMR spectrum (400 MHz, $CDCl_3$) of compound **2a** 23
Figure S 21. ^{13}C{^{1}H} NMR spectrum (101 MHz, $CDCl_3$) of compound **2a**) 24
Figure S 22. ^{1}H NMR spectrum (270 MHz, $CDCl_3$) of compound **2b** 24
Figure S 23. ^{13}C{^{1}H} NMR spectrum (101 MHz, $CDCl_3$) of compound **2b** 25
Figure S 24. ^{1}H NMR spectrum (270 MHz, $CDCl_3$) of compound **3a** 25
Figure S 25. ^{13}C{^{1}H} NMR spectrum (101 MHz, $CDCl_3$) of compound **3a** 26
Figure S 26. ^{1}H NMR spectrum (400 MHz, $CDCl_3$) of compound **4** 26
Figure S 27. ^{13}C{^{1}H} NMR spectrum (101 MHz, $CDCl_3$) of compound **4** 27

Table S 1 Hydrogen bonding parameters in compounds **2a** and **2b** 4
Table S 2 Hydrogen bond parameters in compound **3a** 5
Table S 3 H bond parameters of compounds **4** and **6** 5
Table S 4 Bond parameters of important C–H…CT interactions 6
Table S 5: Parameters of ring-ring interactions with distances shorter than 4.5Å 7
Table S 6 Calculated interaction energies [kJ/mol] for compounds **2a**, **2b** and **5** 8
Table S 7 Calculated interaction energies [kJ/mol] for compound **3a** 9
Table S 8 Calculated interaction energies [kJ/mol] of compounds **4** and **6** 10
Table S 9 Calculated Interaction Energies (E_{tot} y -30 kJ/mol) for para- [$C_6(SC_6H_4OMe)_4(CN)_2$], VOHFOR 10

1. EXPERIMENTAL SECTION (more detailed description)

A solution of **1** (2.400 g, 7.41 mmol) in THF (75 mL) was treated at -78°C with 1.0 M LDA solution (8.90 mL, 8.90 mmol) with stirring for 45 min. Then, solid Andersen reagent (2.620 g, 8.90 mmol) was added, and with continuous stirring the reaction mixture was gradually warmed to room temperature (20 °C) within 16 h. The obtained suspension was evaporated. The residue was taken up in the minimum amount of ethyl acetate and placed on top of a silica gel column.

A first chromatographic run using ethyl acetate as eluent yielded 6 fractions. Fraction F1 contained unreacted Andersen reagent and other unidentified “organics”. Fraction 2 consisted of unreacted **1** (ca. 1.19 g, ca. 49% recovery). Fraction 3 (60 mg) contained besides further unreacted **1** the di-substituted species **2a,b** as well as the tri-substituted **3a,b** and traces of the tetra-substituted **4**. The first three fractions were discarded. Fraction F4 contained mainly compound **4** together with **3a** and **3b** in relative amounts of ca. 2:1.6:1. An ESI mass spectrum showed the presence of di-, tri- and tetrasubstituted species [$C_{10}H_{10-n}(SOTol)_nFe$], n= 2,3,4. The ^{1}H NMR spectrum of fraction F5 showed a plethora of signals in the Cp region. The ESI-MS showed the presence of di-, tri, tetra- and penta-substituted [$C_{10}H_{10-n}(SOTol)_nFe$], n= 2,3,4,5. Fraction F4 was then re-chromatographed, still using ethyl acetate as eluent. Five fractions were obtained. The first fraction F4.1. contained only “organics” and was discarded. The second fraction F4.2. consisted of large amounts of an unidentified species besides **2a,b** and **3b**. Attempts of isolating **3b** from this mixture met with failure. Fraction F4.3. consisted mainly of compounds **4**, **3a**, and **3b** (rel. 2:2:1) together with several unidentified impurities (again δ ≈ 4.37). The next fraction, F4.4, contained mainly compounds **4** and **3a**, together with small amounts of **3b** and several unidentified species. The last fraction F4.5. contained numerous unidentified peaks in the Cp region, one of which might be assigned to **5**. Fractions F4.4. and F4.5. were re-chromatographed, using different solvents (PE, CH_3CN) and mixtures of them. While this allowed to completely separate compounds **3a** and **4** from fraction F4.4., no conditions were found to separate compound **5** from the other un-identified compounds.

2. HYDROGEN BONDING AND Π-INTERACTIONS

2.1. H…O and H…S interactions

In compound **2a**, O1 and O2 accept both two hydrogen bonds each (three from phenyl H, one from a Cp hydrogen), while sulphur atom S1 accepts one weak H bond from phenyl hydrogen H.

In compound **2b** weak bonds between oxygen atoms O11 and cyclopentadienyl hydrogens H14 join neighbouring ferrocene units. In addition, oxygen atoms O11, O13 and O21 accept (intra and intermolecular) H bonds from phenyl hydrogens (see Table S1).

All oxygen atoms in compound **3a** accept hydrogen bridges, nearly always (except for one) from phenyl CH bonds. Two O atoms, O11 and O23, even accept two H bonds. In addition, sulphur atom S11 also accepts one H bond from phenyl H123 (Table S2).

All oxygen atoms of compound **4** act as H bond acceptors towards phenyl C–H bonds: O101 and O204 accept only one, while the others accept two. O201 additionally accepts one H bond from a Cp C–H bond. About half of them are of intramolecular nature (Table S3).

Apparently, there are no hydrogen bonds in compound **5**, while there are two in compound **6**. In the latter, the two "middle" sulphur atoms act as H bond acceptors towards two phenyl C–H bonds. These H bonds join the individual molecules in *b* direction.

Table S 1 Hydrogen bonding parameters in compounds **2a** and **2b**

Comp.	D-H…A	D–H	H…A	D…A	D–H…A
**2a**	C3-H3…O1^{i}	0.93	2.26	3.182(5)	169
	C13-H13…O2^{ii}	0.93	2.46	3.270/5)	151
	C22-H22…O2	0.93	2.53	2.899(6)	104
	C15-H15…O1^{iii}	*0.93*	*2.62*	*3.519(6)*	*163*
	C23-H23…S1^{iv}	*0.93*	*3.10*	*3.892(5)*	*163*
**2b**	C14-H14…O11^{v}	0.93	2.50	3.349(6)	152
	C116-H116…O11	0.93	2.49	2.889(6)	106
	C132-H132…O13	0.93	2.54	2.927(6)	105
	C212-H212…O21^{vi}	*0.93*	*2.64*	*3.399(6)*	*140*

H bonds as determined by PLATON (normal font) or MERCURY (italics), using standard settings of both programs symm. ops: i) *x-1, y, z;* ii)*: 2-x, ½ +y, 1-z;* iii) *2-x, ½ +y, -z;* iv) *2-x, -½ +y, 1-z;* v) *x, y-1,z;*vi) *x,1-y,z*

Table S 2 Hydrogen bond parameters in compound **3a**

D-H…A	D–H	H…A	D…A	D–H…A
C23-H23…O13^{i}	0.95	2.41	3.152(6)	135
C113-H113…O11^{ii}	0.95	2.19	3.136(6)	173
C116-H116…O11	0.95	2.52	2.921(6)	105
C223-H223…O21^{iii}	0.95	2.22	3.105(5)	154
C225-H225…O22^{i}	0.95	2.43	3.295(1)	151
C232-H232…O23	0.95	2.51	2.912(6)	105
C235-H235…O23^{i}	0.95	2.25	3.189(5)	170
C236-H236…O13^{i}	0.95	2.46	3.364(5)	158
C125-H125…O12^{ii}	0.95	*2.64*	*3.550(5)*	*162*
C123-H123…S11^{iii}	0.95	*2.95*	*3.844(4)*	*157*

H bonds as determined by PLATON (normal font) or MERCURY (italics), using standard settings of both programs symmop: i) *1+x, y, z;* ii) *x-1,y,z*; iii) *x,y-1,z*

Table S 3 H bond parameters of compounds **4** and **6**

Comp.	D-H…A	D–H	H…A	D…A	D–H…A
**4**	C117 -- H11C ... O101^{i}	0.98	2.38	3.253(12)	148
	C247 -- H24B ... O204^{ii}	0.98	2.47	3.428(12)	164
	C110 -- H110 ... O301	0.95	2.48	3.269(16)	140
	C112 -- H112 ... O101	0.95	2.44	2.869(10)	107
	C123 -- H123 ... O102^{i}	0.95	2.43	3.288(10)	150
	C126 -- H126 ... O102	0.95	2.49	2.899(10)	106
	C132 -- H132 ... O104	0.95	2.50	3.384(10)	156
	C133 -- H133 ... O103^{i}	0.95	2.42	3.348(10)	167
	C136 -- H136 ... O103	0.95	2.46	2.877(10)	106
	C143 -- H143 ... O104^{iii}	0.95	2.47	3.282(11)	143
	C145 -- H145 ... O301^{i}	0.95	2.38	3.323(12)	171
	C208 -- H208 ... O401^{v}	0.95	2.55	3.47(5)	163
	C209 -- H209 ... O201^{iv}	0.95	2.50	3.434(12)	167

	C212 -- H212 ... O201^{iv}	0.95	2.34	3.243(12)	159
	C222 -- H222 ... O201	0.95	2.38	3.256(9)	153
	C223 -- H223 ... O202^{ii}	0.95	2.36	3.281(10)	164
	C226 -- H226 ... O202	0.95	2.51	2.909(10)	106
	C236 -- H236 ... O203	0.95	2.53	2.930(10)	105
	C246 -- H246 ... O204	0.95	2.42	2.846(11)	107
	C233-H233…O203^{i}	0.95	2.69	3.426(1)	135
**6**	C34-H34…S2^{i}	0.95	2.86	3.702(2)	148
	C36-H36…S3^{vi}	0.95	2.94	3.736(2)	143

H bonds as determined by PLATON (normal font) or MERCURY (italics), using standard settings of both programs symmop: i) *x, y-1, z;* ii*) x, 1+y, z;* iii)*: 1-x,½ +y,-z;* iv) *1-x,y-½,1-z;* v*) -x, ,y-½,1-z;* vi)*: -x,1-y,-z*

2.2. C–H…C and C…C interactions

Although there are several "isolated" C–H…C and C…C interactions between molecules (compare for these the Figures related to the individual contributions to the interaction energies further down), we want to concentrate here on interactions between C–H bonds and π-rings and between two different π systems. Analysis was performed using PLATON.

While there are apparently no interactions of the C–H…π type for compounds **2a** and **5**, there are three for compound **2b**, two for compound **3a**, nine for compound **4** and three for compound **6** (Table S4).

Table S 4 Bond parameters of important C–H…CT interactions

Comp	C–H…CT	H…CT [Å]	C–H–CT [°]
**2b**	C117-H11B…CT(C211-C216)^{i}	2.54	159
	C137–H13C…CT(C25b-C29b)^{ii}	2.85	141
	C237-H22A…CT(C131-C136)^{iii}	2.92	135
**3a**	C126-H126…CT(C10-C14)	2.81	135
	C226-H226…CT(C20-C24)	2.73	135
**4**	C113-H113…CT(C231-C236)	2.76	127
	C116-H116…CT(C121-C126)	2.67	151

	C122-H122…CT(C131-C136)	2.64	148
	C125-H125…CT(241-C246)^{i}	2.73	137
	C216-H216…CT(C206-C210)^{iv}	2.80	154
	C232-H232…CT(C221-C226)	2.54	157
	C235-H235…CT(C121-C126)^{iii}	2.64	143
	C242-H242…CT(C231-C236)	2.64	140
	C403-H43B…CT(C211-C216)^{v}	2.93	150
**6**	C5-H5…CT(C21-C26)^{i}	2.65	169
	C25-H25…CT(C41-C46)^{ii}	2.89	120
	C46-H46…CT(C31-C36)$_{i}$	2.79	146

Symmop: i) *1+x, y, z*; ii) *1-x, ½ +y, 1-z*; iii) *x-1, y, z*; iv) *x, 1+y, z*; v) *-x, ½ +y, 1-z*

Table S 5: Parameters of ring-ring interactions with distances shorter than 4.5Å

Comp	CT(Ring 1)…CT(Ring 2)	R [Å]	α[°]	ß[°]	γ[°]
**2a**	CT(C11-C16)…CT(C21-C26)	4.156(3)	32.3(2)	33.8	3.2
**3a**	CT(C111-C116)…CT(C121-C126)	3.700(3)	6.4(2)	22.0	25.5
	CT(C111-C116)…CT(C211-C216)^{i}	4.298(3)	12.7(2)	32.6	29.6
	CT(C121-C126)…CT(C131-C136)	3.780(3)	14.1(2)	23.2	19.2
	CT(C131-C136)…CT(C231-C236)^{ii}	3.821(3)	2.5(2)	27.2	25.4
	CT(C211-C216)…CT(C221-C226)	3.897(3)	6.4(2)	30.3	28.2
	CT(C221-C226)…CT(C231-C236)	3.749(3)	11.8(2)	28.9	18.1

Symmop: i) *x-1, y, z-1*; ii) *x-1, 1+y, z:*

α =∠(CT(Ring 1), CT(Ring 2)); ß= ∠ CT(1)→CT(2) and normal to plane (ring 1); γ =∠ Cg(I)→Cg(J) vector and normal to plane (ring 2)

3. INTERACTION ENERGIES

Interaction energies were calculated using the program TONTO, as provided within the *CrystalExplorer* program suite. Although this program allows the use of the more exact CE-B3LYP/6-31G(d,p), we used the much faster HF/3-21G method due to restrictions in computer time. The program output contains a set of individual interactions listed according to the distances R between interacting molecule centers, together with the individual energy contributions of electronic (E_{el}), polarization (E_{pol}), dispersion (E_{dis}) and repulsion (E_{rep}) terms to the total energies E_{tot}. For the present discussion, only contributions with $|E_{tot}| > 10$ kJ/mol were considered. The results are shown in Tables S6-S8 and Figures S11-S16 of the Supporting Information. Where two independent molecules were found in the unit cell, only one was selected, as also shown in the Tables. As *CrystalExplorer* unfortunately cannot handle disordered molecules, the disordered solvent in **4** had to be excluded from the calculations, and only one of the disordered C_5H_5 rings in **2b** was selected.

For the disubstituted compounds six (**2a**), nine (**2b**) and seven (**5**) interactions were found with $E_{tot} < -10$ kJ/mol. The largest interaction energy was found for an H…O interaction with compound **2a**, and for H…C interactions with compounds **2b** and **5**, For all compounds and all individual types, the dispersion term was the most important, reaching -92.3 kJ/mol for interaction type A of compound **2b**. However, in some cases the electronic term was nearly as important (C, E, F for compound **2b**).

Eight interactions were found for compound **3a** to have $E_{tot} < -10$ kJ/ mol. The largest value of -65.0 kJ/mol was found for a combination of H…H, H…O and H…S interactions. Again, for all individual types the contribution of the dispersion term was the largest, reaching -64.3 kJ/mol for type A, while for type B the contributions of E_{ele} and E_{disp} were nearly identical.

In the tetrasubstituted compounds, nine interactions with $E_{tot} < -10$ kJ/mol were found for both **4** and **6**. The largest interaction energies were found twice for compound **4**, amounting to -84.9 kJ/mol both for a combination of H…H and H…C interactions as well for a combination of H…C and H…S interactions. A special situation with compound **4** was the presence of ethyl acetate molecules in the lattice. Still, five out of the nine selected interaction types involve the ordered ethyl acetate. Once again, for both compounds and all types of interactions the dispersion term was the dominant one; however, in contrast to the less substituted compounds discussed above, the electronic terms are always much smaller.

3.1. Disubstituted Compounds

Table S 6 Calculated interaction energies [kJ/mol] for compounds **2a**, **2b** and **5**

Type/ Comp	Symmop[a]	R [Å]	E_{ele}	E_{pol}	E_{dis}	E_{rep}	E_{tot}	Important Interactions

**2a**								
A	*x+1, y, z*	7.90	-22.8	-12.9	-32.8	20.8	-44.2	H...O
B	*2-x, y-½,-z*	8.43	-5.3	-4.2	-53.5	20.3	-39.8	H...C, H...S
C	*2-x, y-½,1-z*	8.68	-12.4	-8.4	-36.9	20.6	-34.6	H...H, H...C, H...O, H...S
D	*2-x, y+½,-z*	10.27	-13.3	-7.2	-20.4	9.2	-29.2	H...C
E	*x, y+1, z*	12.91	-0.3	-1.9	-15.9	0	-15.9	H...C
F	*1-x, y-½,1-z*	9.94	-2.0	-1.0	-22.4	8.7	-15.8	H...S
**2b**/ Mol. B								
A	A: *x-1, y, z*	8.90	-21.6	-6.7	-92.3	50.0	-68.9	H...C
B	B: *x, y-1, z*	6.04	-18.3	-8.6	-55.7	25.3	-53.9	H...H, H...C, H...O
C	B: *-x, y+½, 1-z*	8.93	-26.0	-14.4	-29.6	22.1	-44.6	H...H, H...C, H...O, H...S, S...O
D	A: *x-1, y-1, z*	9.10	-7.4	-3.3	-46.2	17.6	-37.0	H...H, H...C
E	A: *x, y, z*	9.50	-16.3	-7.0	-22.9	9.0	-34.5	H...C, H...O, H...S, O...C
F	A: *-x, y-½,-z*	12.72	-10.7	-1.6	-13.4	0.0	-24.1	H...C
G	A: *1-x, y-½, 1-z*	12.58	-0.9	-1.6	-14.3	0.0	-14.9	H...C
H	A: *x, y-1, z*	9.78	2.9	-4.6	-16.2	5.0	-10.6	H...O, H...S
I	B: *-x, y+½,-z*	12.4	10.4	-2.0	-21.5	0.0	-10.1	H...C
**5**								
A	*x, y, 1+z*	8.85	-8.4	-5.3	-49.2	21.8	-38.7	H...C
B	*x, y-1, z*	7.16	-9.6	-2.1	-31.9	17.2	-26.0	H...C, C...C
C	*1.5-x, -y, z-½*	9.38	-8.7	-2.3	-24.1	12.7	-21.8	H...C
D	*1.5-x, -y, z+½*	9.40	-8.7	-2.3	-24.1	12.7	-21.8	H...C
E	*x- ½, 1-y, z+½*	8.78	-9.7	-1.7	-24.2	15.5	-20.3	H...C, H...S
F	*1.5-x, 1-y, z-½*	8.79	-9.7	-1.7	-24.2	15.5	-20.3	H...C, H...S
G	*x, 1+y, 1+z*	11.38	-0.1	-2.1	-22.6	12.7	-11.6	H...H, H...C

[a] In compound **2b** molecule B was selected for the calculation. The prefixes A and B in the second column hint to which molecule was involved in this particular interaction.

3.2. Tri-substituted compounds

Table S 7 Calculated interaction energies [kJ/mol] for compound **3a**

Type/	Symmop[a]	R [Å]	E_{ele}	E_{pol}	E_{dis}	E_{rep}	E_{tot}	Important Interactions
**3a**/ Mol.A								
A	A: *1+x, y, z*	7.83	-27.3	-16.8	-64.3	39.2	-65.0	H...H, H...O, H...S
B	B: *x-1, y, z*	8.98	-38.0	-16.8	-41.1	29.2	-63.0	H...C, H...O, H...S
C	B: *x-1, y, z+1*	10.15	-11.6	-10.9	-42.5	21,8	-39.6	H...O, C...O, C...S
D	B: *x-1, y+1, z*	11.05	-12.8	-4.2	-42.6	20.6	-37.5	H...O, H...C, C...C
E	B: *x-1, y+1, z+1*	11.15	-9.0	-3.1	-44.7	20.2	-35.1	H...C
F	A: *x, y-1, z*	9.86	0.8	-4.7	-47.7	23.0	-26.6	H...C, H...S
G	A: *1+x, y-1, z*	13.42	5.0	-2.3	-18.8	0.0	-13.4	H...C, C...C
H	B: *x, y, z*	10.99	4.1	-6.9	-21.5	9.8	-11.7	H...O, C...O

[a] In compound **3a** molecule A was selected for the calculation. The prefixes A and B in the second column hint to which molecule was involved in this particular interaction

3.3. Tetra-substituted compounds

Table S 8 Calculated interaction energies [kJ/mol] of compounds **4** and **6**

Type/	Symmop[a]	R [Å]	E_{ele}	E_{pol}	E_{dis}	E_{rep}	E_{tot}	Important Interactions
**4**/ Mol.A								
A	A: *x, y-1, z*	8.22	-43.0	-24.2	-82.0	59.8	-84.9	H…H, H…C, H…O, H…S
B	B: *x, y, z*	11.22	-43.0	-24.2	-82.0	59.8	-84.9	H…H, H…C, H…O, H…S
C	A: *1-x, y-½, -z*	11.47	-23.5	-7.8	-39.6	21.1	-47.5	H…C, H…O
D	EA: *x, y, z*	6.91	-9.1	-5.6	-44.6	27.4	-30.9	H…C, H…H, H…O
E	EA: *1-x, y-½,-z*	7.73	-6.1	-2.8	-16.4	7.5	-16.7	H…O
F	EA: *x, y-1, z*	8.65	-9.8	-4.4	-12.7	10.0	-16.3	H…O
G	A: *1+x, y, z*	12.89	8.7	-1.4	-22.4	0.0	-12.3	H…H
H	EA: *1+x, y-1, z*	9.28	-3.0	-1.1	-15.2	6.6	-12.0	H…C, H…O
I	EA: *1+x, y, z*	7.69	-0.1	-2.8	-20.5	11.7	-11.0	H…C, H…S, H…O, C…O
**6**								
A	*1-x, 1-y, 1-z*	8.85	-19.6	-5.9	-84.9	39.3	-68.4	H…C, C…C
B	*x-1, y, z*	8.48	-19.6	-5.7	-75.4	40.3	-59.0	H…C, H…S
C	*1-x, 1-y, -z*	8.56	-7.9	-5.3	-64.1	43.2	-34.3	H…C, H…S
D	*-x, 1-y, -z*	9.57	-16.9	-4.2	-42.2	34.6	-30.0	H…H, H…C, H…S
E	*1-x, 2-y, 1-z*	11.69	-10.9	-2.2	-35.2	20.8	-27.4	H…H, C…C
F	*x, 1+y, z*	10.30	-10.2	-2.3	-37.8	25.7	-25.1	H…C
G	*-x, -y, -z*	15.03	-5.5	-0.5	-9.6	0.0	-14.6	H…C
H	*2-x, 1-y, 1-z*	11.64	0.4	-1.2	-21.3	7.9	-13.1	H…H, H…C
I	*-x, 1-y, 1-z*	12.85	0.2	-0.5	-11.8	0.0	-10.7	H…C

[a] In compound **4** molecule A was selected for the calculation. The prefixes A and B in the second column hint to which molecule was involved in this particular interaction, including the ordered ethyl acetate (EA)

3.4. The literature Compound VOHFOR

Table S 9 Calculated Interaction Energies (E_{tot} y -30 kJ/mol) for para- [$C_6(SC_6H_4OMe)_4(CN)_2$], VOHFOR

Type/	Symmop[a]	R [Å]	E_{ele}	E_{pol}	E_{dis}	E_{rep}	E_{tot}	Important Interactions
A	*1+x, y, z*	8.49	-38.1	-10.5	-71.0	41.4	-76.1	H…H, H…S, H…N
B	*1-x, -y, 1-z*	9.67	-38.3	-12.4	-55.7	35.8	-68.3	H…C, H…S, H…N, S…C
C	*x, y, 1+z*	10.45	-39.7	-14.1	-58.7	43.0	-67.6	H…H, H…C, H…S, H…N
D	*-x, -y, 1-z*	12.80	-25.2	-6.1	-60.9	0.0	-33.1	H…H, H…C, H…S

4. FIGURES

4.1. Crystallography

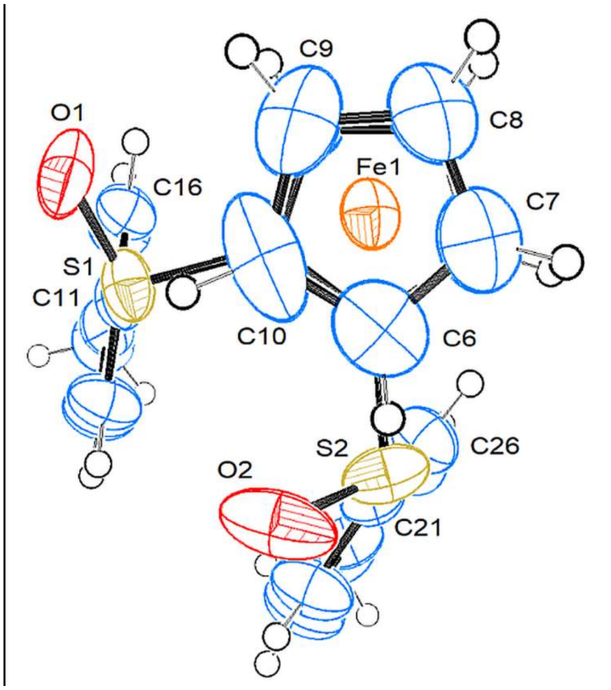

Figure S 1. Molecular structure of compound **2a**, top view.

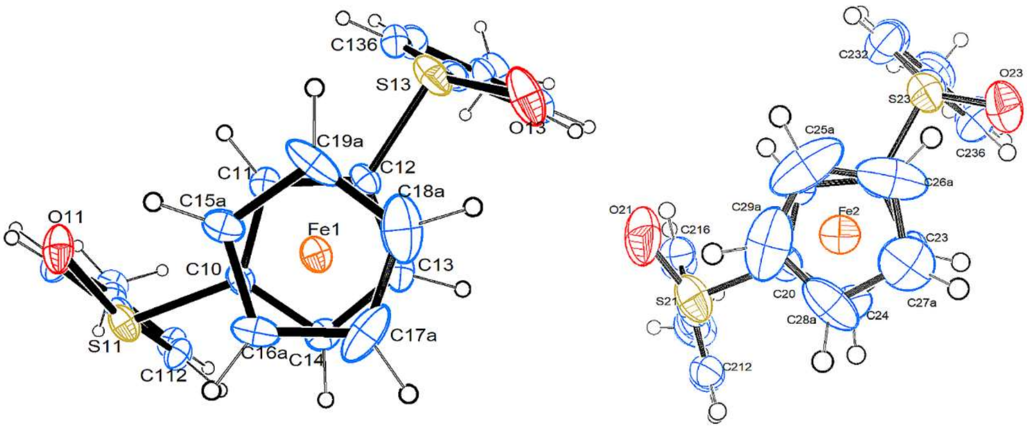

Figure S 2 Molecular structure of compound **3a**, top views of molecules A and B

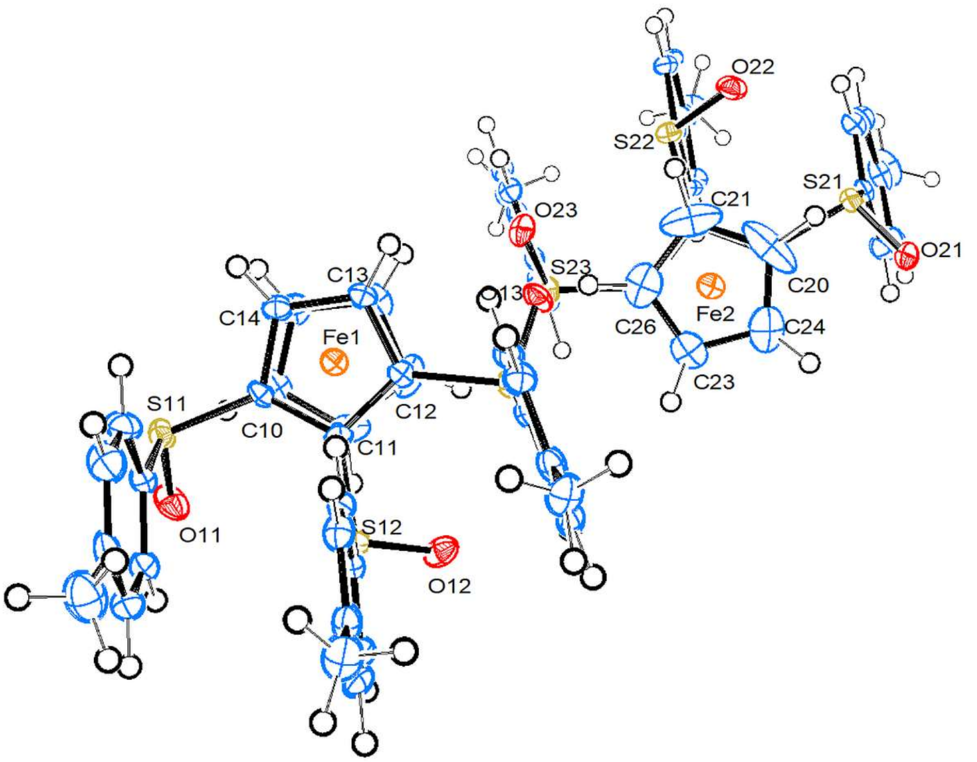

Figure S 3. Molecular structure of compound **3a**, molecules A and B. Top view.

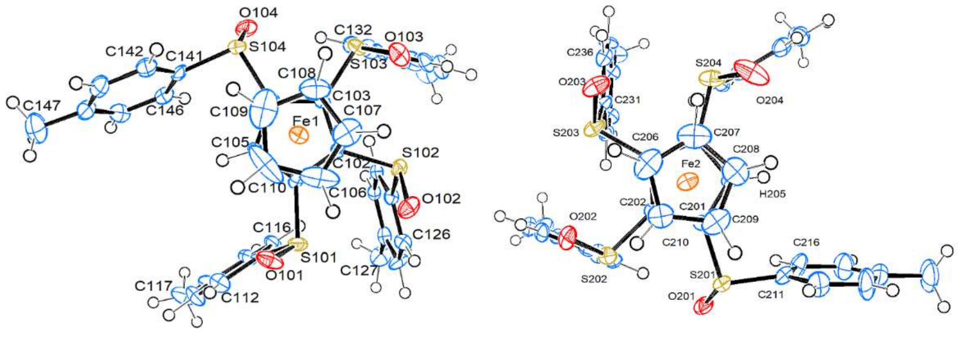

Figure S 4 Molecular structure of compound **4**, top views of molecules A and B

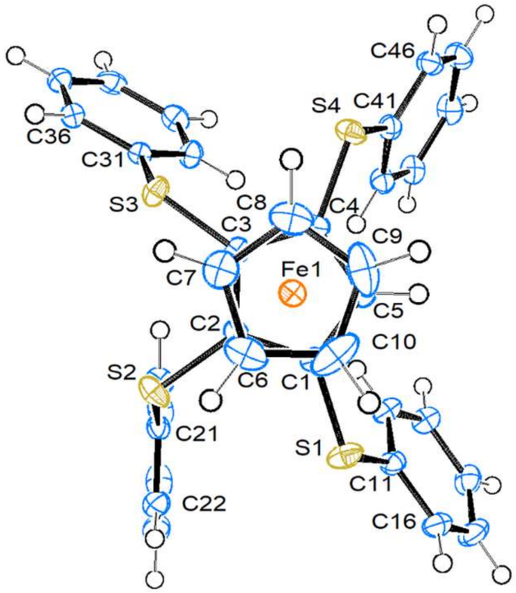

Figure S 5. MOLECULAR STRUCTURE of compound **6**: top view

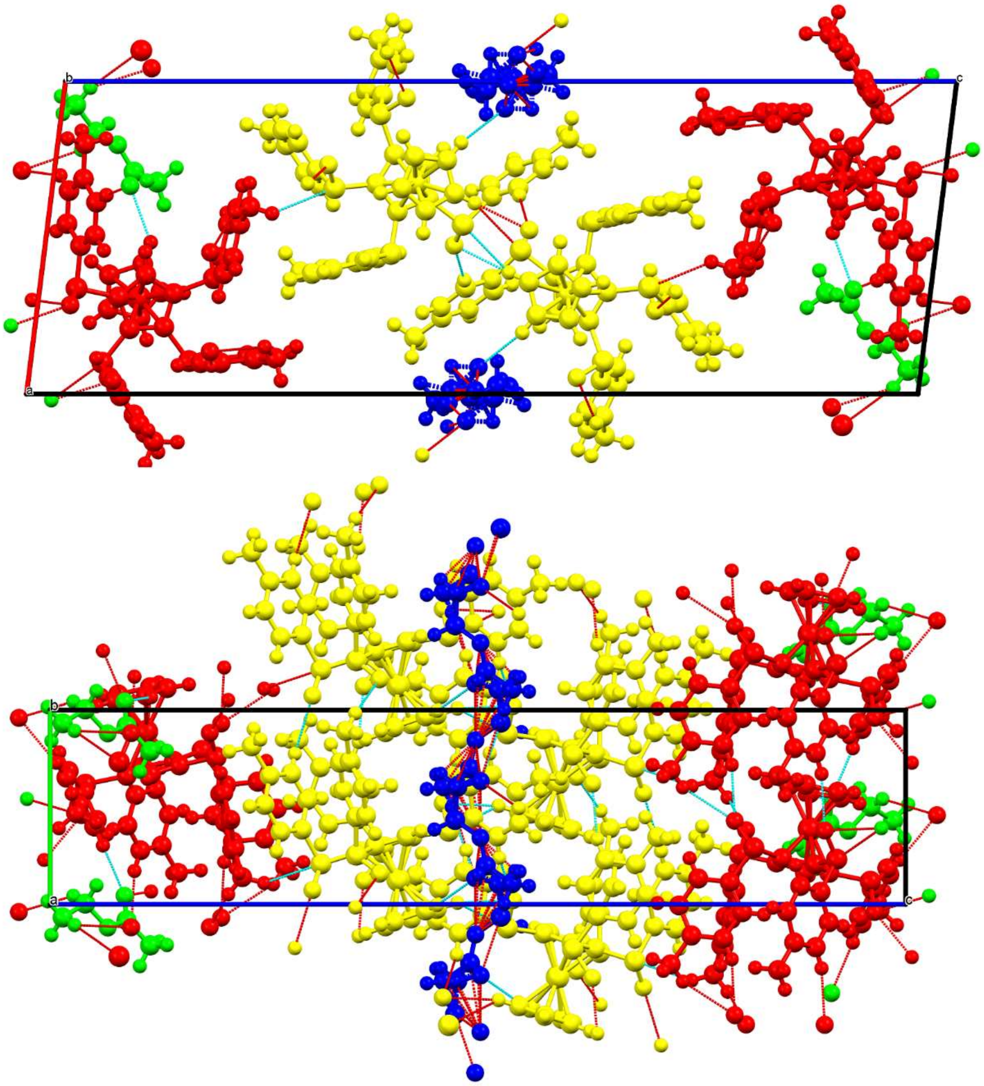

Figure S 6. MERCURY packing plot of compound **4,** using colour coding “by symmetry equivalence”. Red and blue are molecules A and B, respectively, green is the “ordered” ethylacetate and dark blue the “disordered” ethylacetate. Top: viewed along *b*; bottom: viewed along *a*.

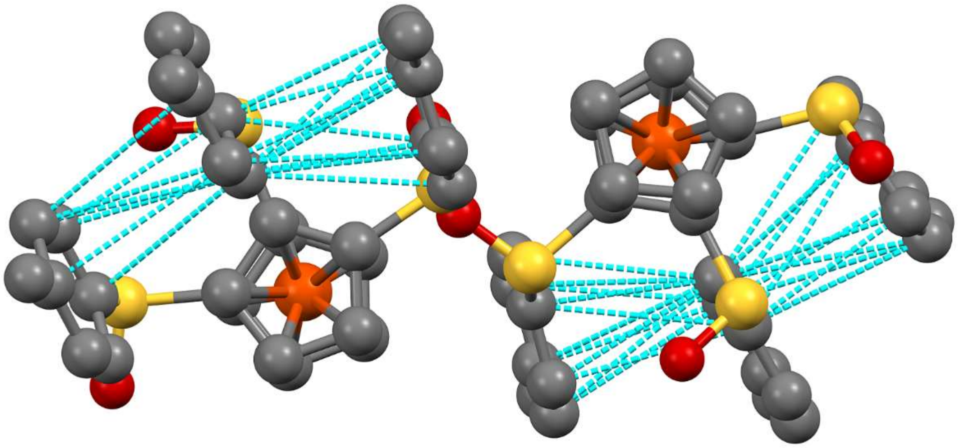

Figure S 7 C…C interactions between the phenyl rings of compound **3a.** Upper limit for the blue dotted lines: van-der-Waals radius plus 0.30 Å. Hydrogen atoms omitted for clarity**.**

4.2. Hirshfeld Analysis

4.2.1. Fingerprint plots

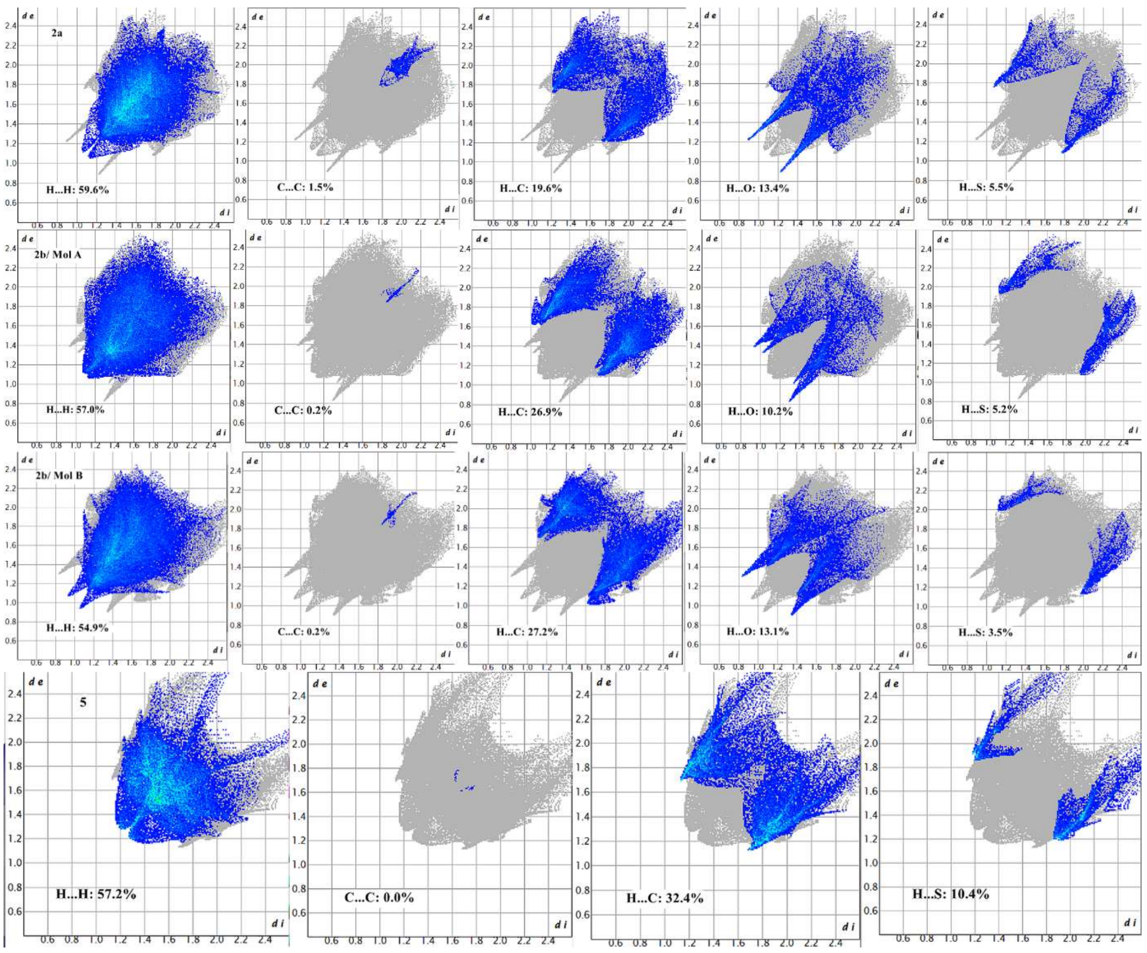

Figure S 8 Individual Fingerprint plots of the disubstituted compounds **2a**, **2b** and **5**

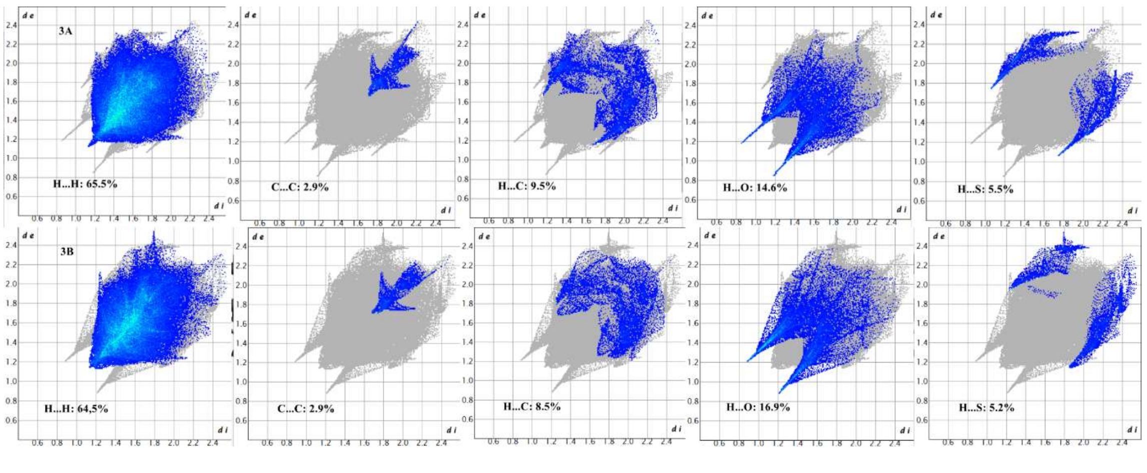

Figure S 9 Individual Fingerprint plots of the tri-substituted compound **3a**

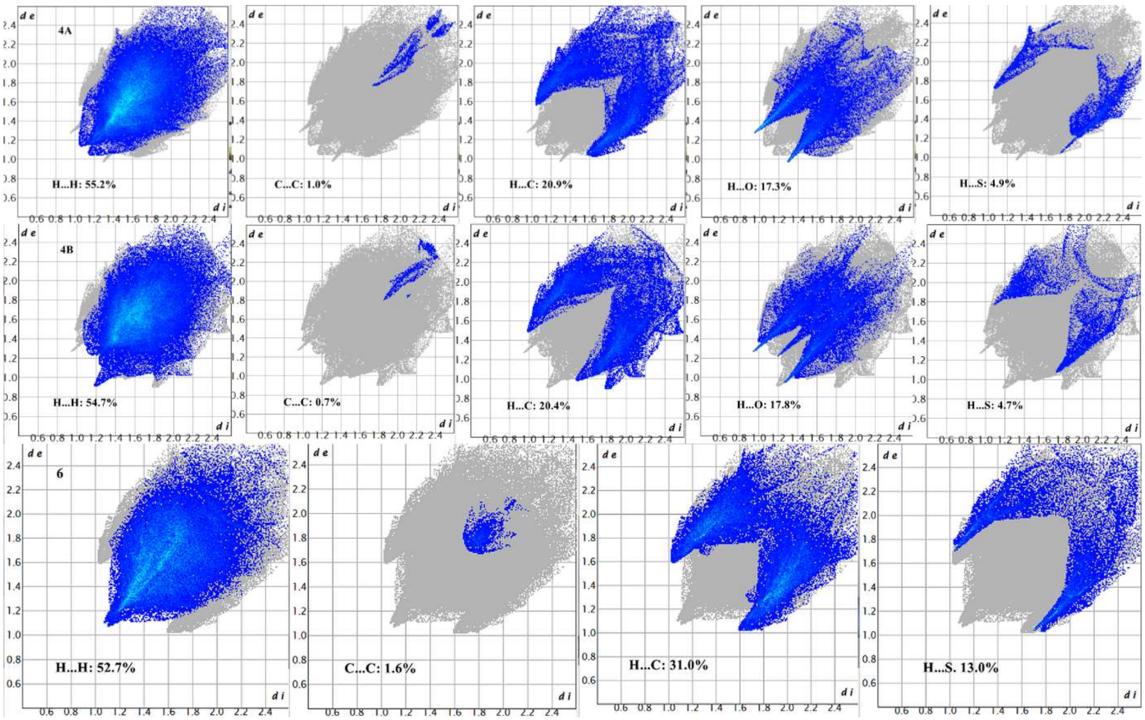

Figure S 10 Individual Fingerprint plots of the tetrasubstituted compounds **4** and **6**.

4.2.2. Interaction Energies: individual contributions (MERCURY standard settings vor close contacts)

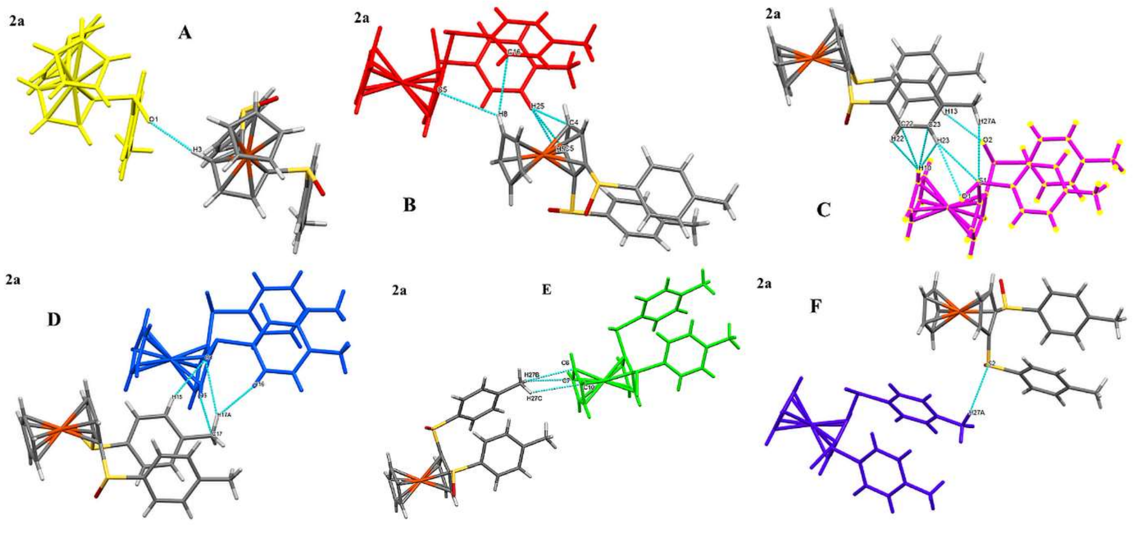

Figure S 11 Individual Contributions A-F to the Interaction Energies of compound **2a**

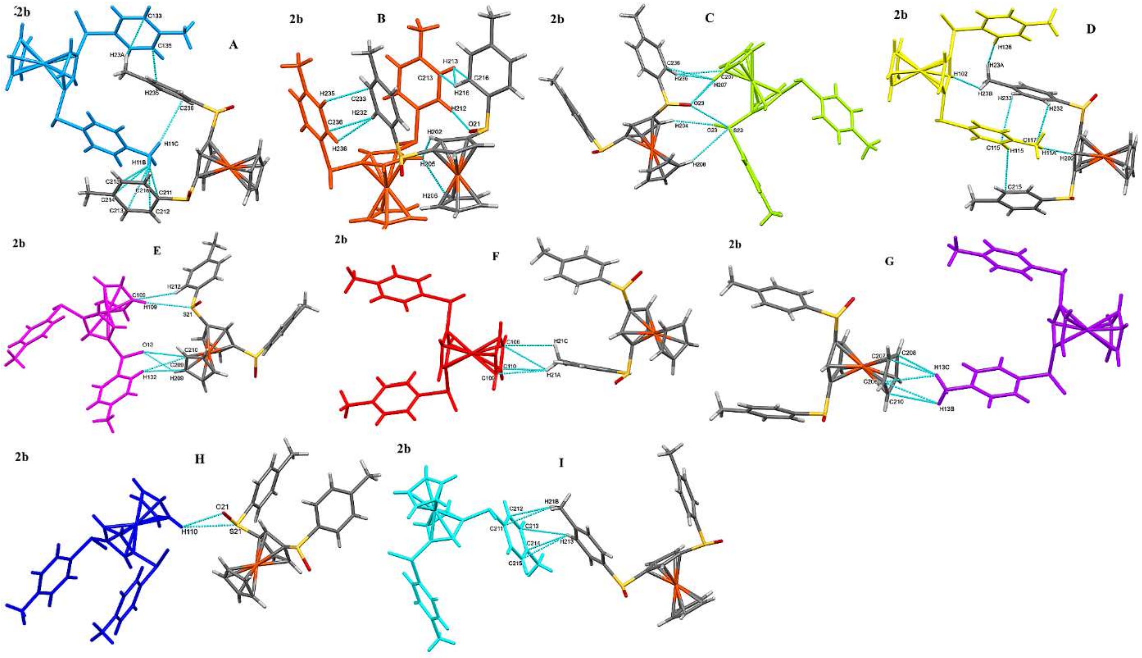

Figure S 12 Individual Contributions A-I to the Interaction Energies of compound **2b**

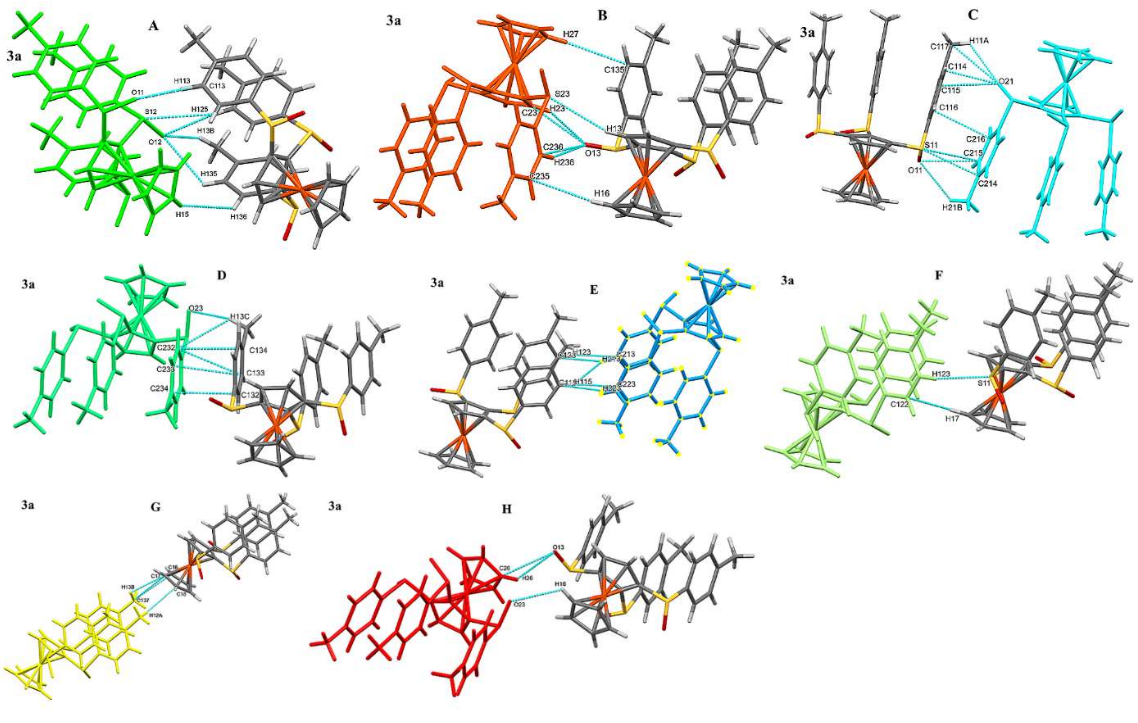

Figure S 13 Individual Contributions A-H to the Interaction Energies of compound **3a**

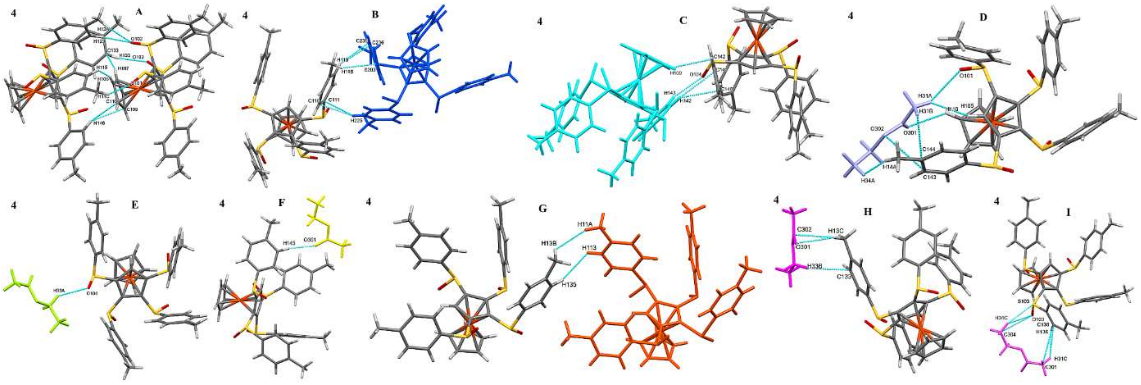

Figure S 14 Individual Contributions A-I to the Interaction Energies of compound **4**

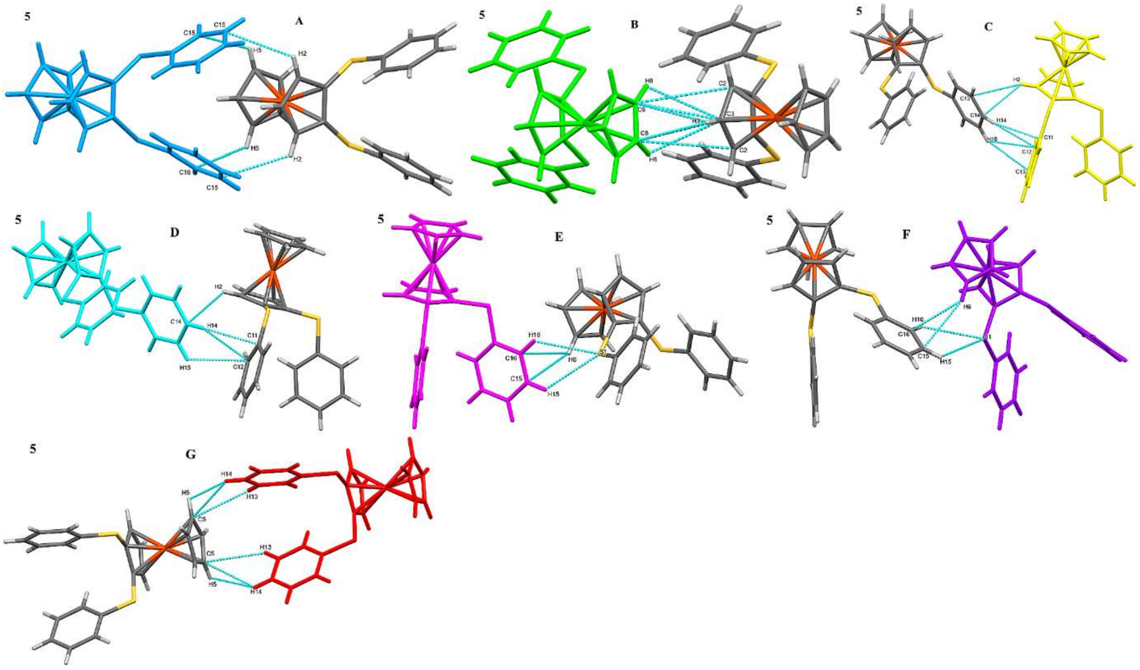

Figure S 15 Individual Contributions A-G to the Interaction Energies of compound **5**

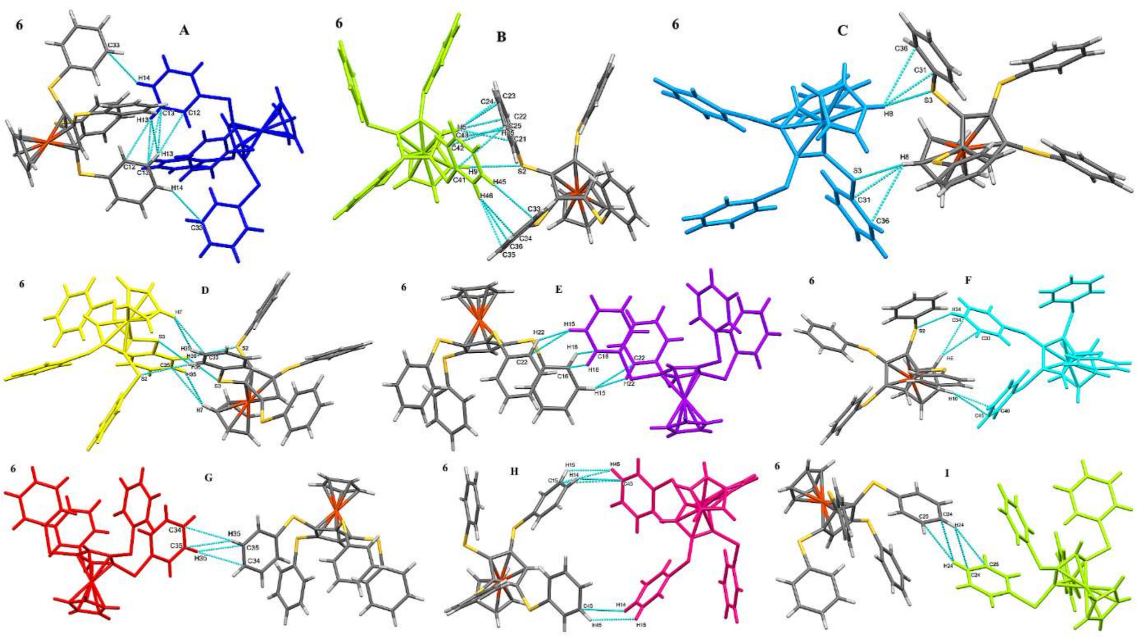

Figure S 16 Individual Contributions A-I to the Interaction Energies of compound **6**

4.2.3. Interaction energies: Selected individual contributions (extended *CrystalExplorer* settings)

4/ A

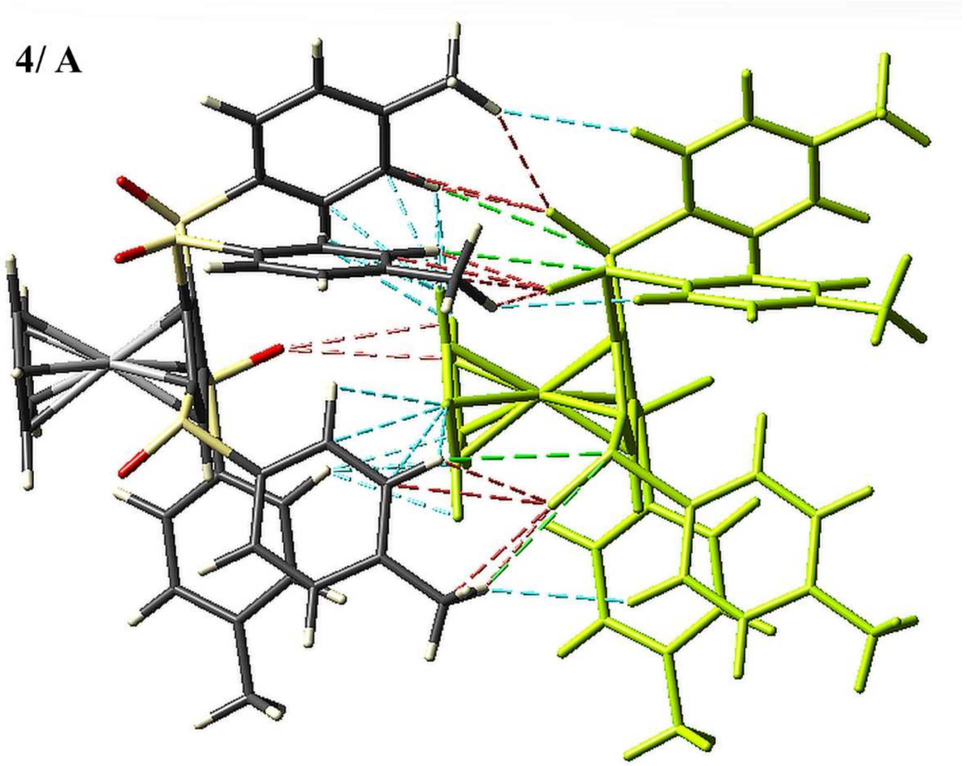

4/ B

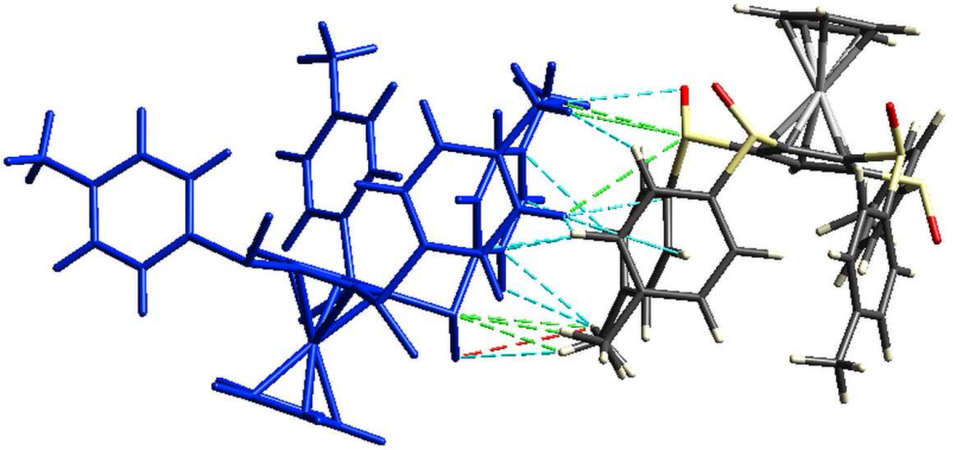

Figure S 17: Interaction Energies for Compound **4**: the two strongest interactions (colour coding: green: S…any, up to 3.80 Å; light blue: H…any, up to 3.09 Å, red: O…any, up to 3.52 Å*)*

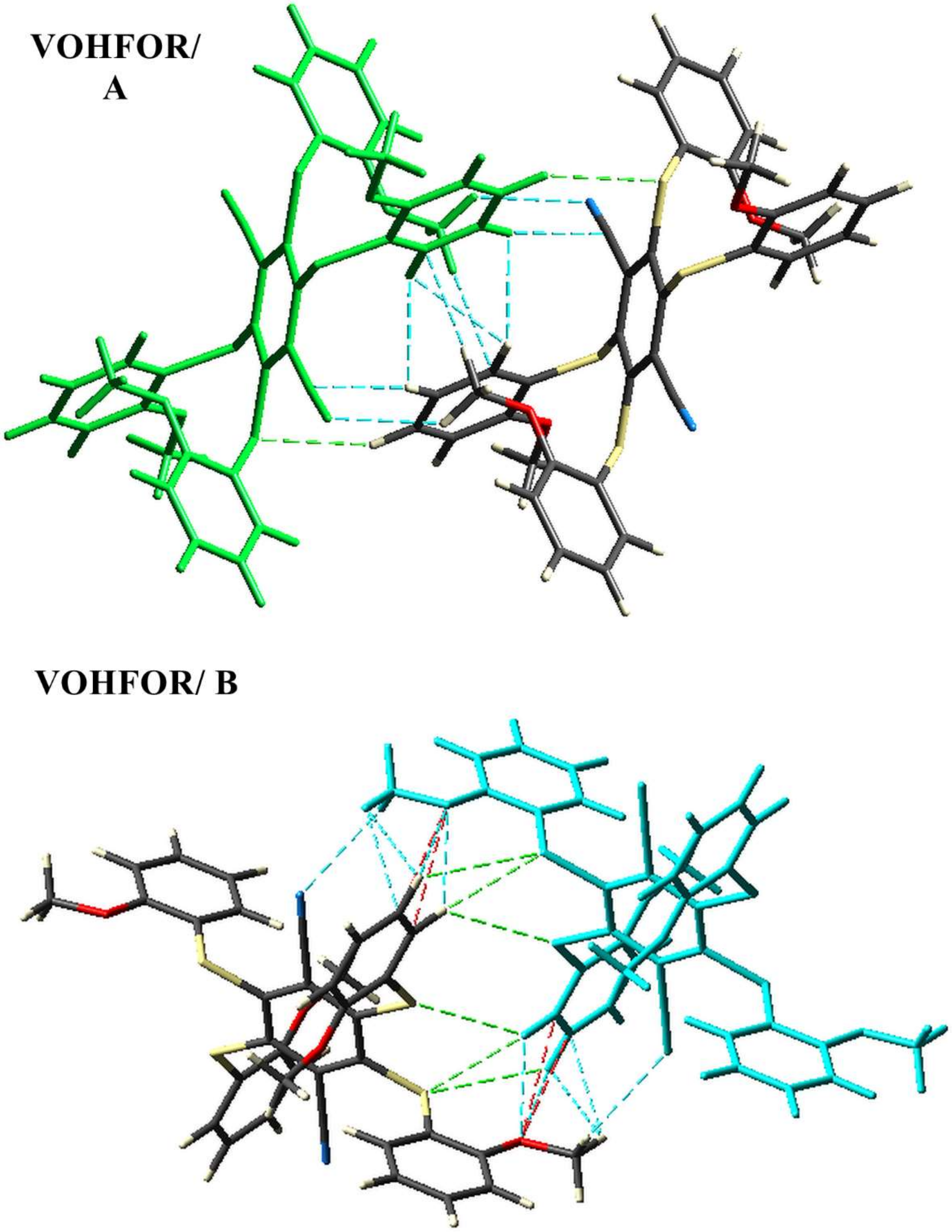

Figure S 18 Interaction Energies for literature Compound **VOHFOR**: the two strongest interactions (colour coding: green: S…any, up to 3.80 Å; light blue: H…any, up to 3.09 Å, red: O…any, up to 3.52 Å*)*

4.3. NMR spectra

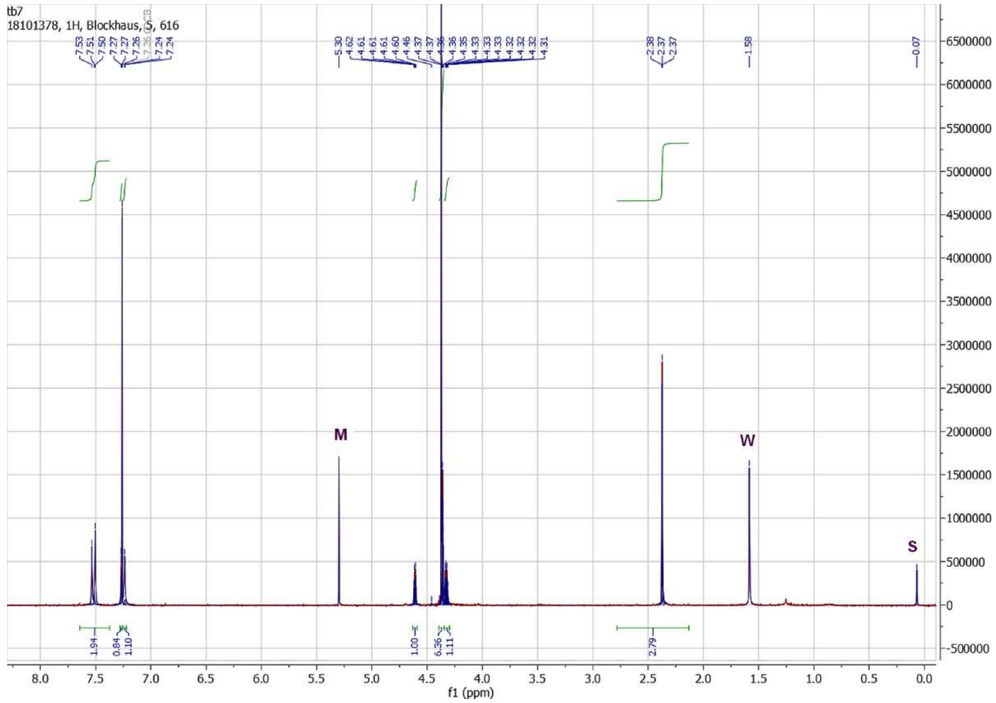

Figure S 19. ^1H NMR spectrum (270 MHz, $CDCl_3$) of compound **1** (M= methylene chloride; W= water; S= silicon grease)

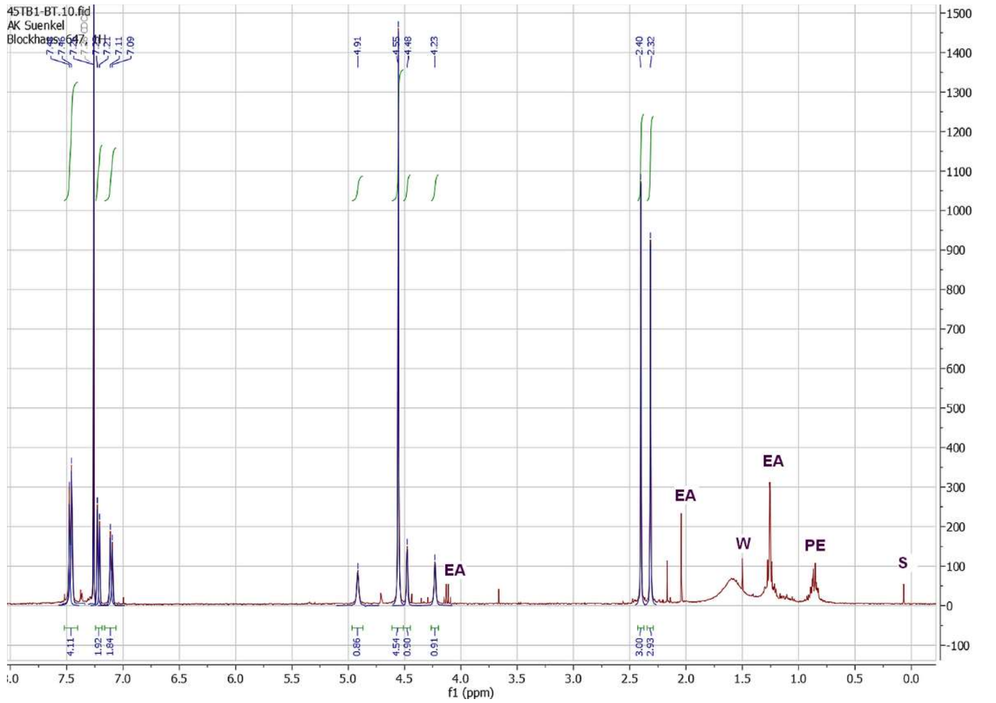

Figure S 20. ^1H NMR spectrum (400 MHz, $CDCl_3$) of compound **2a** (EA= ethyl acetate; PE= petroleum ether)

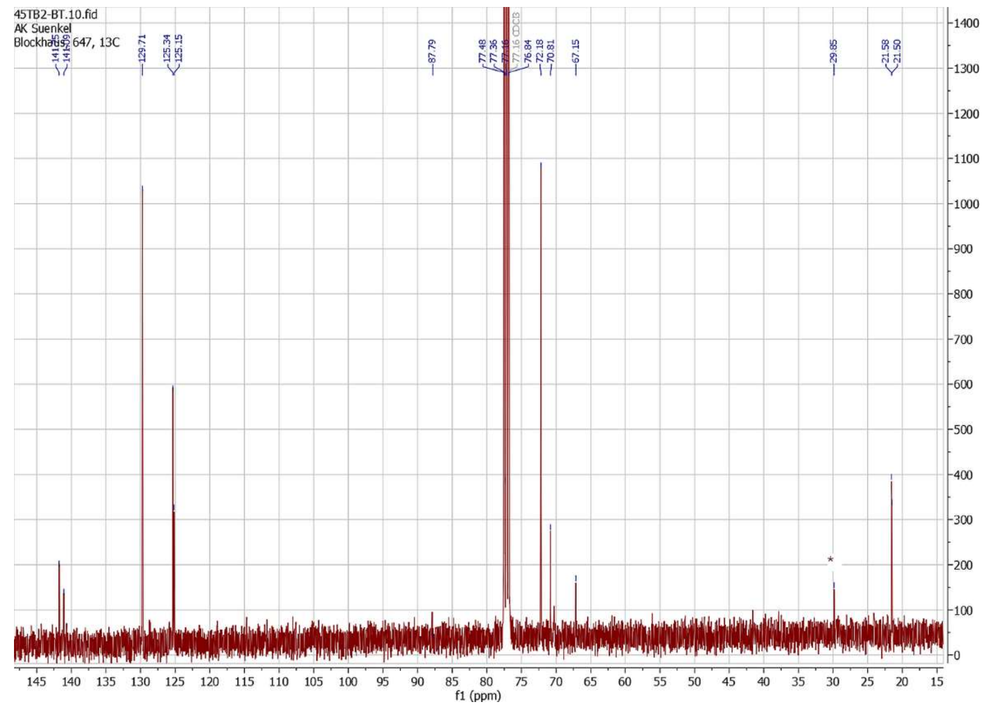

Figure S 21. $^{13}C\{^{1}H\}$ NMR spectrum (101 MHz, $CDCl_3$) of compound **2a** (* unidentified impurity)

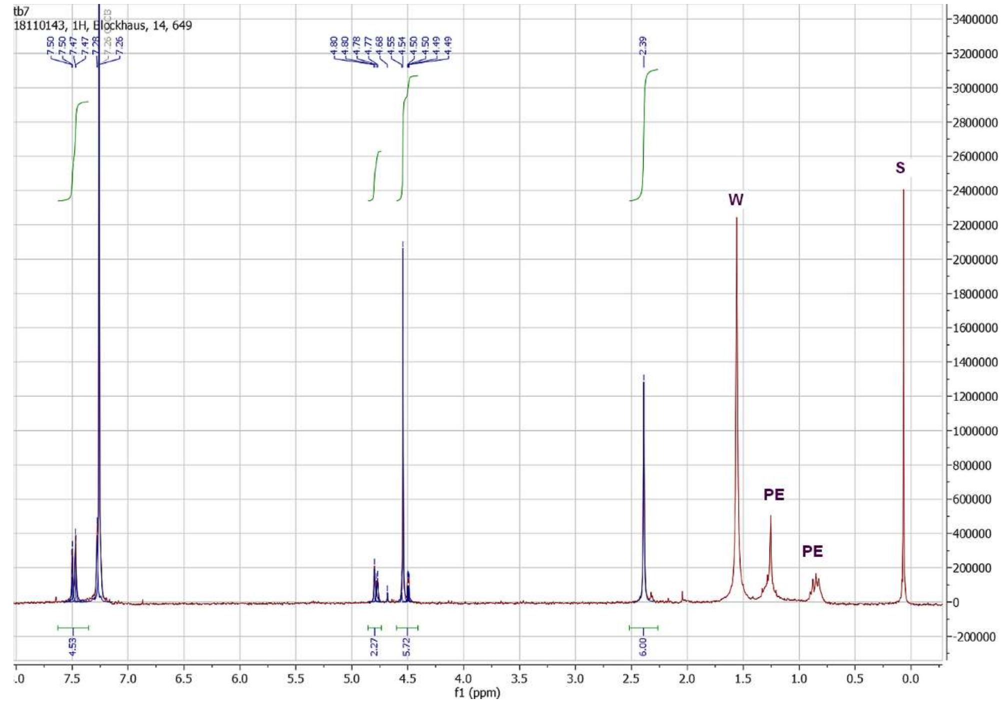

Figure S 22. ^{1}H NMR spectrum (270 MHz, $CDCl_3$) of compound **2b** (impurities marked as defined above*)*

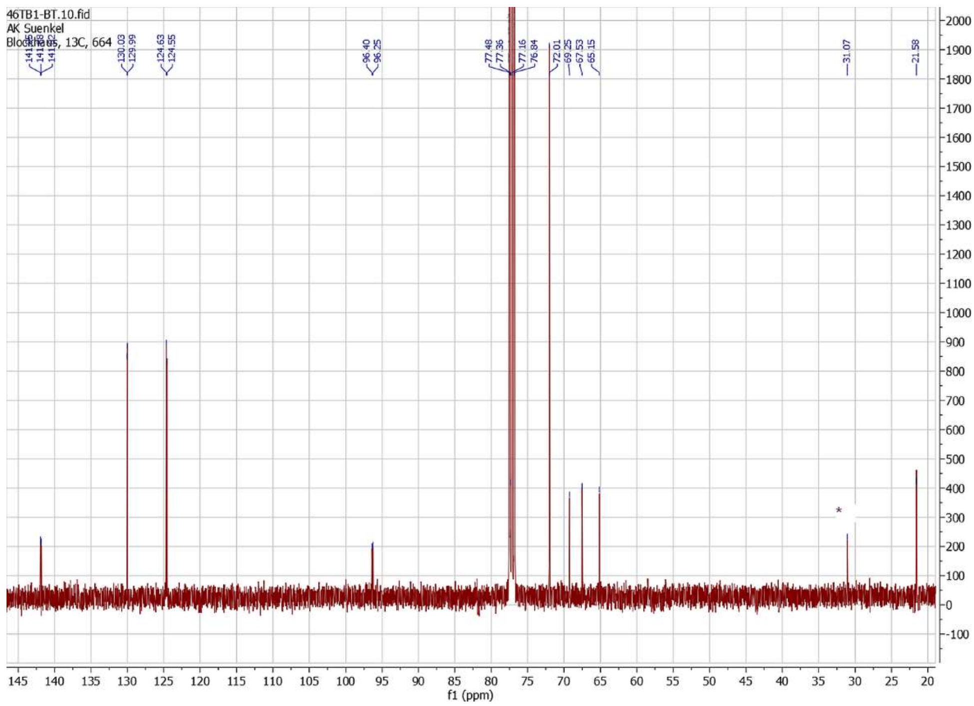

Figure S 23. $^{13}C\{^{1}H\}$ NMR spectrum (101 MHz, $CDCl_3$) of compound **2b** (impurities marked as defined above)

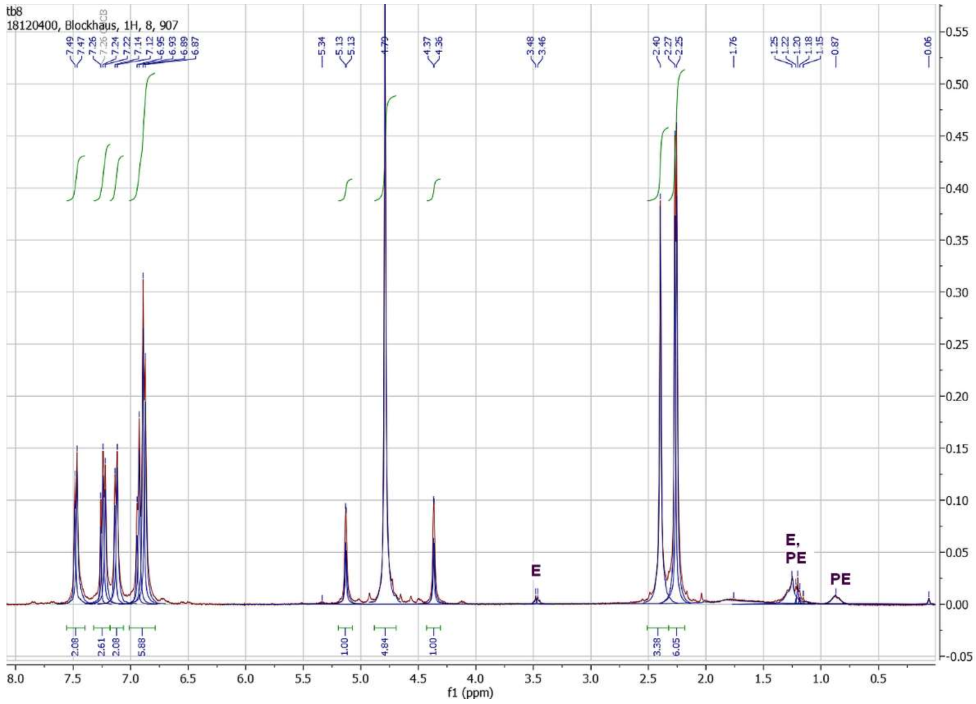

Figure S 24. ^{1}H NMR spectrum (270 MHz, $CDCl_3$) of compound **3a** (E= diethyl ether, PE= petroleum ether)

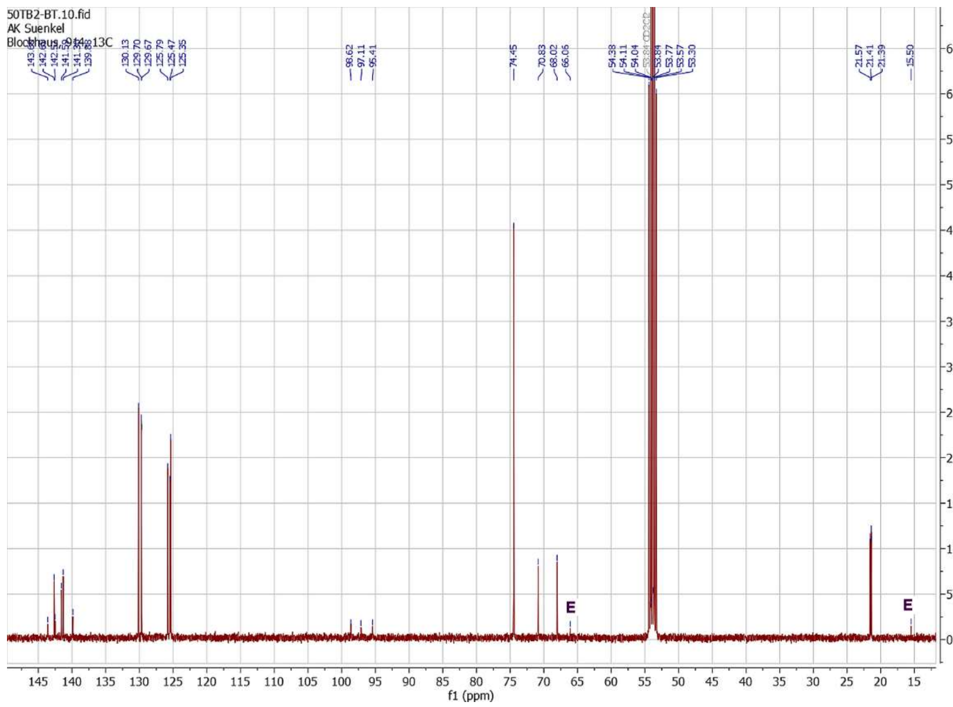

Figure S 25. $^{13}C\{^{1}H\}$ NMR spectrum (101 MHz, $CDCl_3$) of compound **3a** (impurities marked as defined above)

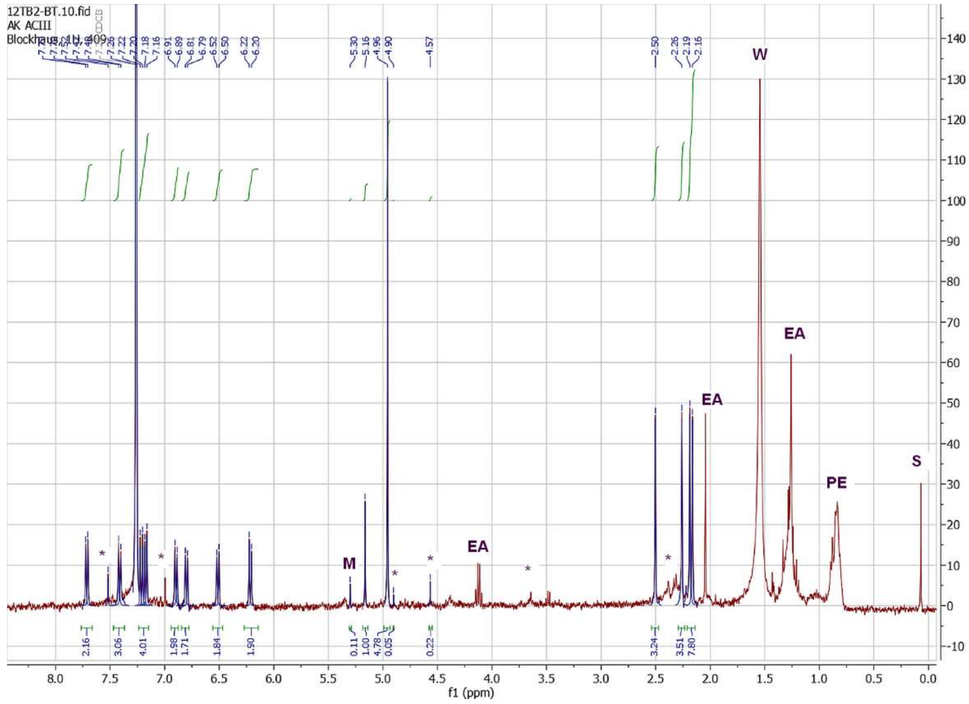

Figure S 26. ^{1}H NMR spectrum (400 MHz, $CDCl_3$) of compound **4** (impurities marked as defined above)

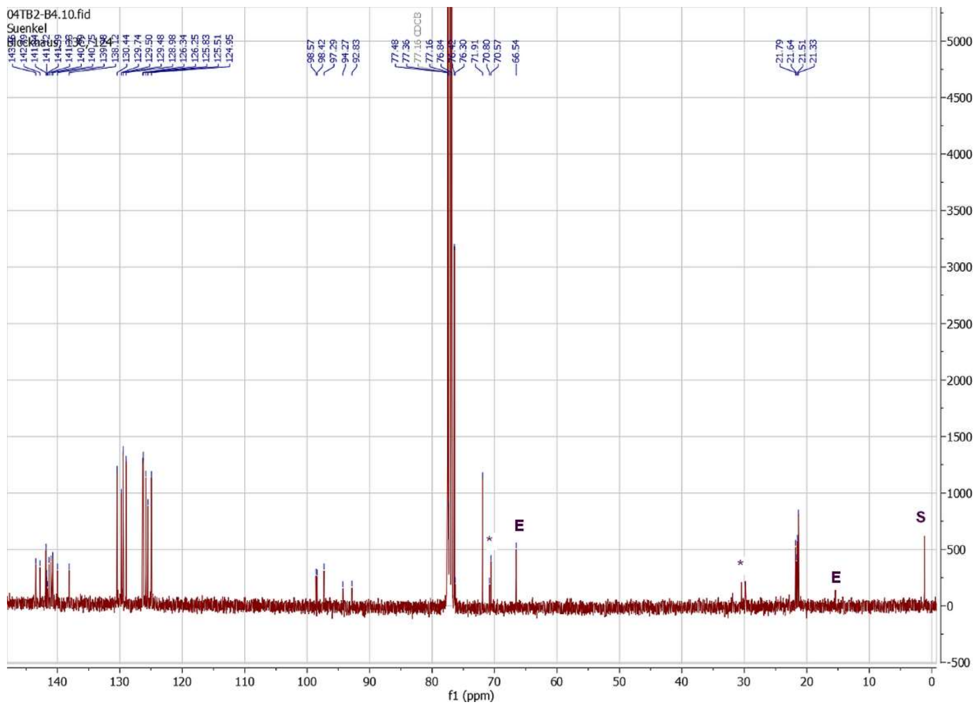

Figure S 27. $^{13}C\{^{1}H\}$ NMR spectrum (101 MHz, $CDCl_3$) of compound **4** (impurities marked as defined above)
